# Optimizing material composition determination in dual-energy computed tomography: a comparative study of a linear model and a fully connected neural network

**DOI:** 10.1093/rpd/ncaf179

**Published:** 2026-03-13

**Authors:** Alexandr Malusek, Sofie Malmodin, Maria Magnusson, Michael Sandborg, Åsa Carlsson Tedgren

**Affiliations:** Department of Health, Medicine and Caring Sciences, Linköping University, SE-581 83 Linköping, Sweden; Center for Medical Image Science and Visualization (CMIV), Linköping University, SE-581 83 Linköping, Sweden; Department of Electrical Engineering, Linköping University, SE-581 83 Linköping, Sweden; Center for Medical Image Science and Visualization (CMIV), Linköping University, SE-581 83 Linköping, Sweden; Department of Electrical Engineering, Linköping University, SE-581 83 Linköping, Sweden; Department of Health, Medicine and Caring Sciences, Linköping University, SE-581 83 Linköping, Sweden; Center for Medical Image Science and Visualization (CMIV), Linköping University, SE-581 83 Linköping, Sweden; Department of Medical Physics, Linköping University Hospital, SE-581 83 Linköping, Sweden; Department of Health, Medicine and Caring Sciences, Linköping University, SE-581 83 Linköping, Sweden; Center for Medical Image Science and Visualization (CMIV), Linköping University, SE-581 83 Linköping, Sweden; Department of Nuclear Medicine and Medical Physics, Karolinska University Hospital, SE-171 76 Stockholm, Sweden

## Abstract

Accurate elemental decomposition in dual-energy computed tomography (DECT) is crucial for precision in radiation therapy planning. We present a comparative study of linear regression and fully connected neural networks (FCNNs) for voxel-wise prediction of tissue elemental composition, using synthetic datasets that incorporate realistic intra- and inter-patient variability. Both models performed well under noise-free conditions, with linear regression yielding slightly lower errors. Under noisy conditions, performance degraded for both models, though the linear model generally retained lower numerical error. The FCNNs, however, consistently produced physically plausible (non-negative) elemental mass-fraction estimates. These models are well suited for integration into model-based iterative reconstruction algorithms to support artificial intelligence-driven radiation treatment planning. Future work should incorporate elemental covariances and spatial context to enhance accuracy and clinical utility.

## 1 Introduction

Accurate knowledge of tissue elemental composition is essential for improving the precision of absorbed dose calculations in radiation therapy. Traditionally, treatment planning systems estimate tissue properties from computed tomography (CT) images by converting Hounsfield values into electron density and tissue type with known elemental composition using empirically derived calibration curves [1–3].

Dual-energy CT (DECT) provides improved accuracy by offering energy-dependent attenuation data [4–6]. This enables material decomposition techniques that estimate not only electron density but also effective atomic number ($Z_{\mathrm{eff}}$) on a voxel-by-voxel basis. The advent of spectral CT systems has further enabled detailed elemental characterization, including direct estimation of specific elemental concentrations, enhancing tissue differentiation.

DECT allows material decomposition using two- and three-material models [7, 8], which approximate tissue composition as linear combinations of base materials with known elemental composition. The accuracy of these methods depends strongly on the choice of base materials; poor selection can lead to significant errors in estimated composition and derived properties. Projection-domain methods such as the Alvarez–Macovski algorithm [9] apply fixed bases (e.g. water and iodine), while image-domain decomposition offers flexibility to adapt base materials to the imaged tissue. Model-based iterative reconstruction algorithms (MBIR), such as DIRA [10], further refine image reconstruction and material decomposition, thereby improving elemental accuracy and reducing beam hardening artefacts.

Data-driven techniques—including statistical reconstruction [11], sparse dictionary-based methods [12], and machine learning [13, 14]—have shown promise in improving decomposition robustness and accuracy. However, these approaches face challenges in managing noise and in ensuring physically valid (non-negative) mass fractions. Negative mass fractions typically arise from the ill-conditioned nature of the system of equations used in material decomposition. Although they may mathematically balance linear attenuation coefficients (LACs), they are incompatible with Monte Carlo dose calculation codes, which require non-negative elemental compositions.

A common limitation of existing methods is their reliance on reference elemental compositions, neglecting natural inter- and intra-patient variability. Variability due to age, sex, pathology, and anatomical location can significantly affect decomposition accuracy if not accounted for.

This study addresses this gap by proposing a method for modelling the variability of elemental tissue composition both within (intra-patient) and among (inter-patient) individuals to generate realistic training data for DECT decomposition of bone and adipose-muscle soft tissues. We use this dataset to train and evaluate both a linear regression model and a fully connected neural network (FCNN) for predicting elemental composition on a voxel-by-voxel basis. The performance of these models is assessed in terms of accuracy, physical plausibility, and robustness under varying noise conditions. This work aims to support future integration into MBIR algorithms, such as DIRA, and to highlight how model-based and artificial intelligence (AI)-based algorithms can complement each other. While AI-based methods offer speed and robustness, model-based algorithms provide high-quality training data and physical interpretability.

## 2 Methods

Computer simulations were conducted to assess the feasibility of estimating tissue elemental composition from DECT data using two modelling approaches: a fully connected (dense) neural network and a linear regression model. Both models were trained to predict elemental mass fractions from monoenergetic LACs at 50 and 88 keV. The training dataset was synthetically generated using reported elemental composition ranges for soft and bone tissues. Model performance and robustness were evaluated across varying noise levels using quantitative error metrics.

### 2.1 Data generation

Elemental mass fractions and mass densities were derived from the tissue composition dataset compiled by Woodard and White [15], which reports values for various human tissues. Two tissue categories were modelled: (i) soft tissue and (ii) bone tissue. The soft tissue category included adipose tissue, muscle tissue, and their linear mixtures, with proportions varying continuously from 0 to 1. Similarly, the bone tissue category consisted of cortical bone, red bone marrow, and their mixtures. In both categories, each component—two pure tissues and a mixture—comprised approximately one-third of the total samples. The elemental composition and mass density of each tissue sample were sampled from either normal distributions (when the standard deviation was available) or uniform distributions (when only a range could be defined based on the numeric representation of the data). For example, in Table 1, the standard deviation of the mass fraction of hydrogen is calculated as $(11.6 - 11.2) / 2 = 0.2$%, since the values for adipose 1 and adipose 3 correspond to the mean (adipose 2) plus or minus 1 SD. In contrast, for cortical bone, where the mean hydrogen mass fraction is 3.4%, the numeric precision of the data defines a uniform range of (3.35%, 3.45%). For each category, three dataset variants were generated: one noise-free and two with added noise corresponding to signal-to-noise ratios (SNRs) of 10 and 5.

**Table 1:** Elemental mass fractions, $w$, and densities, $\rho$, for selected body tissues [15].

Tissue	$w_{\mathrm{H}}$	$w_{\mathrm{C}}$	$w_{\mathrm{N}}$	$w_{\mathrm{O}}$	$w_{\mathrm{Ca}}$	$\rho$
	(per cent)	(per cent)	(per cent)	(per cent)	(per cent)	(kg m$^{-3}$)
Adipose 1	11.2	51.7	1.3	35.5	0.0	970
Adipose 2	11.4	59.8	0.7	27.8	0.0	950
Adipose 3	11.6	68.1	0.2	19.8	0.0	930
Muscle 1	10.1	17.1	3.6	68.1	0.0	1050
Muscle 2	10.2	14.3	3.4	71.0	0.0	1050
Muscle 3	10.2	11.2	3.0	74.5	0.0	1050
Cort. bone	3.4	15.5	4.2	43.5	22.5	1920
Red b. m.	10.5	41.4	3.4	43.9	0.0	1030

For example, the soft tissue category was generated as follows. For a total of $N$ simulated samples, $N/3$ samples were drawn from normal distributions representing the mass fractions of H, C, N, and O for adipose tissue, muscle tissue, and their mixtures. The mixture samples were created by linearly combining adipose and muscle compositions with mixing ratios ranging from 0 to 1. Corresponding mass densities for the mixtures, $\rho _{\textrm{mix}}$, were calculated using the harmonic mean of the component densities


1 \begin{eqnarray*} \rho_{\textrm{mix}} = \left( \frac{w_{1}}{\rho_{1}} + \frac{w_{2}}{\rho_{2}} \right)^{- 1},\end{eqnarray*}

where $w_{1}$ and $w_{2}$ with the normalizing condition $w_{1} + w_{2} = 1$ are the mass fractions of adipose tissue and muscle tissue, respectively, and $\rho _{1}$ and $\rho _{2}$ are the corresponding mass densities. This formulation assumes ideal volumetric mixing, meaning that the mixture volume is taken as the sum of the component volumes. Any samples containing negative mass fractions were discarded using rejection sampling, effectively imposing a non-negativity cut-off on the underlying normal distribution. The datasets for adipose tissue, muscle tissue, and their mixtures were then combined and randomly shuffled. The bone tissue category was generated using the same procedure, with cortical bone and red bone marrow as the component tissues. In this case, however, Ca was added to the set of simulated elements (H, C, N, and O).

For each generated tissue composition, the corresponding LACs at 50 and 88 keV were calculated using the mixture rule


2 \begin{eqnarray*}& \mu(E) = \rho_{\textrm{mix}} \sum_{i}^{} w_{i} \mu_{\textrm{m},i}(E),\end{eqnarray*}

where $\rho _{\textrm{mix}}$ is the mass density of the mixture calculated from equation (1), $w_{i}$ is the mass fraction of the element $i$, and $\mu _{\textrm{m},i}(E)$ is the mass attenuation coefficient of the element $i$ at energy $E$. Data for mass attenuation coefficients were obtained from the EPDL 97 cross-section data library [16].

Gaussian noise corresponding to SNRs of 10 and 5 was added to the calculated LACs to generate two additional datasets per tissue category. The SNR was defined as


3 \begin{eqnarray*}& \textrm{SNR} = \frac{\mu}{\sigma_\mu},\end{eqnarray*}

where $\mu$ is the LAC and $\sigma _\mu$ is the standard deviation of the added noise.

All input features were standardized using z-score normalization to facilitate model training and improve model performance.

In total, six datasets were constructed: $D_{1}$–$D_{3}$ for soft tissue mixtures, with noise levels of 0 ($D_{1}$), SNR = 10 ($D_{2}$), and SNR = 5 ($D_{3}$), and $D_{4}$–$D_{6}$ for bone tissue mixtures, with identical noise levels ($D_{4}$: noise-free, $D_{5}$: SNR = 10, $D_{6}$: SNR = 5).

Each tissue sample in these datasets included two LAC values (at 50 and 88 keV) and corresponding ground-truth elemental mass fractions. The datasets were divided into training and test sets in a 9:1 ratio, with 20% of the training portion reserved for validation. Each dataset consisted of 10 000 samples.

The entire procedure was repeated 10 times using random-number-generator seeds $0,\ldots ,9$. Standard uncertainties were obtained through a Type A evaluation [17] based on the sample standard deviation of the ten estimates.

### 2.2 Model architectures

#### 2.2.1 Dense neural network

An FCNN without hidden layers (a single linear layer followed by a softmax output) was implemented. The input layer received two standardized (z-score normalized) LAC values at the photon energies of 50 and 88 keV. The output layer predicted either four elemental mass fractions (H, C, N, O) for soft tissue or five (H, C, N, O, Ca) for bone tissue. A softmax activation function was applied to enforce non-negativity and unit-sum constraints on the predicted mass fractions $\widehat{w}_{i}$:


4 \begin{eqnarray*}& \widehat{w}_{i} = \frac{e^{z_{i}}}{\sum_{j=1}^{N} e^{z}_{j}},\end{eqnarray*}

where the pre-activation values are given by


5 \begin{eqnarray*}& \mathbf{z} = \mathbf{W} \mathbf{x} + \mathbf{b}.\end{eqnarray*}

Here, $\mathbf{W} \in \mathbb{R}^{K \times 2}$ and $\mathbf{b} \in \mathbb{R}^{K}$ are the trainable weights and biases, respectively, while $\mathbf{x} = [\tilde{\mu }_{1}, \tilde{\mu }_{2}]^{T} \in \mathbb{R}^{2}$ contains the standardized LAC values. The number of output elements is $K = 4$ for soft tissue and $K = 5$ for bone tissue. For each element $i$, the pre-activation is


6 \begin{eqnarray*}& z_{i} = W_{i,1} \tilde{\mu}_{1} + W_{i,2} \tilde{\mu}_{2} + b_{i}\end{eqnarray*}

Thus, the network contains $K(2 + 1)$ trainable parameters: $4 \times 3 = 12$ parameters for soft tissue and $5 \times 3 = 15$ parameters for bone tissue. A schematic illustration of the network architecture is provided in the supplementary material.

The network was trained using the mean squared error (MSE) loss function and the Adam optimizer [18], with a learning rate of 0.001, a batch size of 16, and 80 training epochs. Mean absolute error (MAE) and MSE were recorded for monitoring convergence.

#### 2.2.2 Linear regression

For comparison, a standard linear regression model was fitted independently for each element using the form


7 \begin{eqnarray*}& {\widehat{w}}_{i} = \beta_{0} + \beta_{1}\tilde{\mu}_{1} + \beta_{2}\tilde{\mu}_{2} + \epsilon,\end{eqnarray*}

where ${\widehat{w}}_{i}$ is the predicted mass fraction, $\tilde{\mu }_{1}$ and $\tilde{\mu }_{2}$ are the standardized LACs at 50 and 88 keV, respectively, $\beta _{0}$, $\beta _{1}$, and $\beta _{2}$ are model coefficients, and $\epsilon$ is the residual error. Each element-specific model, therefore, contains three parameters. Across all elements, the linear models include 12 parameters for soft tissue and 15 parameters for bone tissue, matching the parameter counts of the neural network models. The key distinction is that linear regression can produce any real-valued outputs, whereas the neural network’s softmax activation restricts predictions to non-negative values that sum to one, ensuring physically consistent elemental mass fractions.

### 2.3 Model evaluation

Model performance was evaluated on the test set using the root mean squared error (RMSE):


8 \begin{eqnarray*}& \textrm{RMSE} = \sqrt{\frac{1}{N}\sum_{j = 1}^{N}\left( {\widehat{w}}_{j} - w_{j} \right)^{2}},\end{eqnarray*}

where $N$ is the number of test samples, ${\widehat{w}}_{j}$ is the predicted mass fraction, and $w_{j}$ is the corresponding ground truth.

### 2.4 Software and hardware

Data generation, preprocessing, and linear regression were conducted in MATLAB R2024b. Neural network models were developed in Python 3 using the PyTorch framework. All computations were executed on a workstation equipped with an Intel^®^ Xeon^®^ E-2224G processor (3.50 GHz, 64 GB RAM). An NVIDIA GeForce RTX 3080 Ti GPU (12 GB VRAM) was used to accelerate both the training and evaluation of the neural network models.

## 3 Results

**Figure 1:**
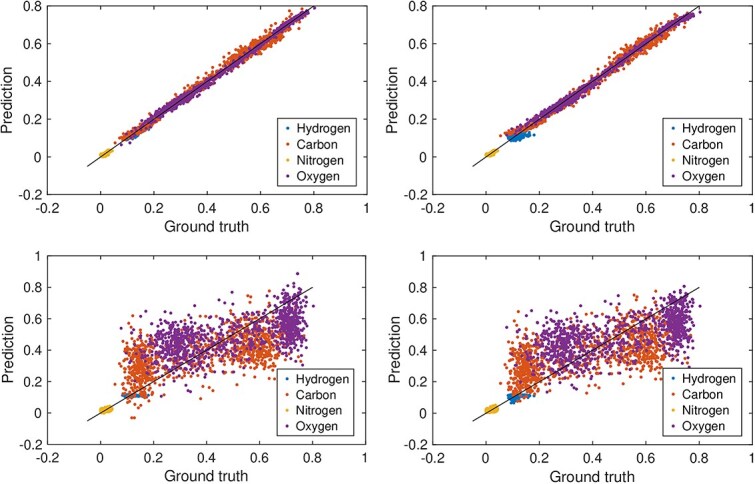
Predicted versus true elemental mass fractions for soft tissue from the linear regression model (left column) and neural network model (right column), shown for data without added noise (top row) and with an SNR of 10 (bottom row).

**Figure 2:**
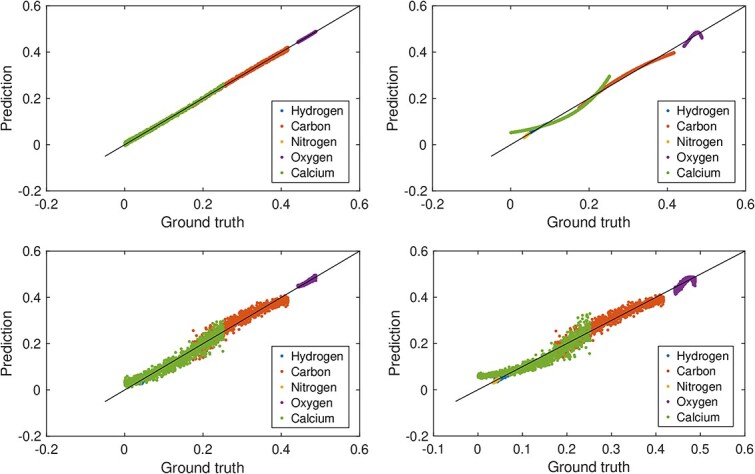
Predicted versus true elemental mass fractions for bone tissue from the linear regression model (left column) and neural network model (right column), shown for data without added noise (top row) and with an SNR of 10 (bottom row).

The neural network converged in fewer than 20 epochs. The results presented correspond to epoch 80, by which point the model had stabilized. Figures 1 and 2 display scatter plots comparing the predictions of the neural network and the linear model to the ground truth for soft tissue and bone tissue, respectively. The diagonal line represents perfect agreement between predictions and ground truth; proximity of data points to this line indicates higher predictive accuracy.

Under noise-free conditions, the linear model produced highly accurate predictions for both tissue types. The neural network exhibited a slight curvature relative to the diagonal but still agreed well with the ground truth. Because the network consists of a single affine transformation followed by a softmax activation, this curvature reflects the non-linearity introduced by the softmax rather than any learned non-linear relationship in the data. At an SNR of 10, noise degraded the predictions of both models. For bone tissue, the predicted values remained comparatively close to the diagonal, whereas for soft tissue, the scatter increased markedly, indicating reduced predictive precision. These trends align with the RMSE values in Tables 2 and 3, where the linear model generally outperformed the neural network, apart from the prediction of carbon in bone tissue.

**Table 2:** RMSE for soft tissue predictions by the linear model (LM) and neural network (NN) on test data, with values scaled by a factor of $10^{3}$ for readability.

	No noise		SNR = 10		SNR = 5	
	NN	LM	NN	LM	NN	LM
H	11.13 $\pm$ 0.05	7.41 $\pm$ 0.09	12.33 $\pm$ 0.09	10.74 $\pm$ 0.13	11.25 $\pm$ 0.15	10.98 $\pm$ 0.15
C	21.15 $\pm$ 0.18	19.07 $\pm$ 0.23	163.63 $\pm$ 1.29	164.22 $\pm$ 1.25	193.88 $\pm$ 0.37	193.64 $\pm$ 0.38
N	4.31 $\pm$ 0.03	4.39 $\pm$ 0.02	9.75 $\pm$ 0.09	9.59 $\pm$ 0.09	11.20 $\pm$ 0.03	11.13 $\pm$ 0.03
O	13.27 $\pm$ 0.14	11.71 $\pm$ 0.13	160.19 $\pm$ 1.24	160.95 $\pm$ 1.20	189.23 $\pm$ 0.39	189.10 $\pm$ 0.40

**Table 3:** RMSE for bone tissue predictions by the linear model (LM) and neural network (NN) on test data, with values scaled by a factor of $10^{3}$ for readability.

	No noise		SNR = 10		SNR = 5	
	NN	LM	NN	LM	NN	LM
H	3.92 $\pm$ 0.07	1.242 $\pm$ 0.006	6.01 $\pm$ 0.03	5.46 $\pm$ 0.04	9.3 $\pm$ 0.1	8.91 $\pm$ 0.08
C	8.61 $\pm$ 0.07	4.269 $\pm$ 0.020	18.82 $\pm$ 0.12	19.63 $\pm$ 0.12	31.4 $\pm$ 0.3	32.01 $\pm$ 0.31
N	1.99 $\pm$ 0.05	0.326 $\pm$ 0.001	2.44 $\pm$ 0.07	1.07 $\pm$ 0.01	3.0 $\pm$ 0.1	1.71 $\pm$ 0.02
O	9.75 $\pm$ 0.18	0.847 $\pm$ 0.004	10.47 $\pm$ 0.08	3.69 $\pm$ 0.02	10.6 $\pm$ 0.1	6.01 $\pm$ 0.06
Ca	22.33 $\pm$ 0.10	4.459 $\pm$ 0.022	29.29 $\pm$ 0.14	20.36 $\pm$ 0.13	39.2 $\pm$ 0.3	33.22 $\pm$ 0.32

The fit quality of the linear model, expressed as adjusted $R^{2}$, is presented in Table 4. In the absence of noise, adjusted $R^{2}$ values exceeded 0.99 for all elements except hydrogen and nitrogen in soft tissue, which showed values of 0.56 and 0.86, respectively.

**Table 4:** Adjusted $R^{2}$ values for bone and soft tissue fits using the linear model, where an SNR of $\infty$ indicates data without added noise, and values are scaled by a factor of $10^{3}$ for readability.

	Bone tissue			Soft tissue		
SNR:	$\infty$	10	5	$\infty$	10	5
H	995.95 $\pm$ 0.02	923.6 $\pm$ 0.4	787.5 $\pm$ 0.6	563.68 $\pm$ 2.77	62 $\pm$ 2	21.7 $\pm$ 0.9
C	996.33 $\pm$ 0.02	923.8 $\pm$ 0.4	787.6 $\pm$ 0.6	991.65 $\pm$ 0.07	367 $\pm$ 3	128.6 $\pm$ 1.6
N	992.21 $\pm$ 0.03	919.8 $\pm$ 0.4	784.1 $\pm$ 0.8	864.94 $\pm$ 0.60	340 $\pm$ 2	118.1 $\pm$ 1.9
O	995.86 $\pm$ 0.03	923.5 $\pm$ 0.4	787.3 $\pm$ 0.7	996.72 $\pm$ 0.03	361 $\pm$ 3	126.5 $\pm$ 1.5
Ca	996.28 $\pm$ 0.02	923.8 $\pm$ 0.4	787.6 $\pm$ 0.6			

## 4 Discussion

This study presents a novel approach to modelling intra- and inter-patient variability in elemental tissue composition for DECT applications. By generating synthetic datasets that reflect realistic variability, we trained and evaluated both linear and neural network models for elemental decomposition. To our knowledge, earlier work has not explicitly incorporated both levels of elemental variability when generating synthetic DECT training data.

Both models performed well under noise-free conditions, with linear regression achieving a slightly lower RMSE, particularly for bone tissue. Under noisy conditions, performance declined, especially for soft tissue, reflecting greater ambiguity in decomposing low-contrast structures. It is important to note that the models developed in this work are primarily designed for MBIR algorithms. These algorithms can operate on low-noise data when generating training datasets for AI-based reconstruction, with noise characteristics added in a controlled post-processing phase. Therefore, the high sensitivity of the studied models to noise is not a major limitation for this purpose. Interestingly, the neural network demonstrated enhanced robustness for specific elements, such as carbon in bone, under noisy conditions, suggesting potential advantages in modelling nonlinear relationships.

The reduced $R^{2}$ values for hydrogen and nitrogen may be due to their relatively narrow dynamic range in soft tissues, reflecting limited inter- and intra-tissue variability in their mass fractions (Table 1), which makes them more difficult to predict from the LACs. Nevertheless, the influence of these elements on attenuation or energy-deposition calculations is expected to be modest, as their mass fractions are comparatively low.

Equations (4) and (5) share the same mathematical form as those used in multinomial logistic regression, which typically operates on one-hot-encoded categorical targets and is trained with a cross-entropy loss. In our case, however, the network predicts continuous elemental mass fractions $w_{i} \in [0,1]$, not discrete class labels, so the multinomial logistic regression framework is not directly applicable. We therefore used a mean squared error loss, although future extensions could incorporate physics-informed terms reflecting known relationships between attenuation coefficients and elemental composition.

Both the linear model and the neural network used $3K$ parameters, where $K$ is the number of predicted elemental mass fractions. For the linear model, these parameters were obtained almost instantaneously using closed-form analytical expressions. In contrast, determining the neural network parameters required iterative optimization with the Adam algorithm and took $\sim$1 min. Once trained, however, the neural network produced predictions quickly.

Repeated runs with different random-number seeds showed that the linear model exhibited substantially lower parameter variability than the neural network. The corresponding data are provided in the supplementary material. Increasing the number of training samples by a factor of 10 and extending the training from 80 to 500 epochs reduced—but did not eliminate—the variability in the neural network’s weights and biases. This behaviour is consistent with previous findings indicating that stochastic-gradient-based optimizers, including Adam, often fail to converge to global minima and instead tend to settle in non-optimal local minima with high probability [19]. In addition, the softmax activation enforces a normalized output, coupling all components through the denominator. When combined with an MSE loss, this coupling produces biased or poorly conditioned gradients because an error in any single target propagates to all output channels. This limitation has been noted in both early and modern literature on neural network training [20]. Nevertheless, even solutions associated with local minima can yield predictions of sufficient accuracy for the present application.

A key strength of our approach lies in its ability to produce physically plausible outputs. The neural network’s architecture enforced non-negativity and mass fraction normalization, avoiding the unphysical negative estimates occasionally produced by linear regression. This enhances clinical relevance, as physically meaningful predictions are critical for applications in radiation dosimetry and treatment planning.

Tissues consist of various components—such as water, proteins, and lipids in muscle tissue—and changes in one component (e.g. hydration status) affect the mass fractions of multiple elements (e.g. hydrogen and oxygen). This implies that elemental mass fractions exhibit non-zero covariances. However, our model did not incorporate these covariances due to the lack of consistent datasets describing such relationships. Developing representative datasets that capture these dependencies remains a significant challenge.

Additional limitations arise from the dataset’s tissue composition, which was generated from two base tissues mixed in linearly increasing proportions—a distribution that may not fully capture clinical variability. A more representative strategy would be to derive voxel-wise composition statistics from segmented organs, thereby better reflecting anatomical heterogeneity. Another limitation is that elements with low mass fractions contributed only weakly to the loss during neural network training, which may have reduced accuracy for trace constituents. The analysis was also restricted to single-pixel data, preventing the model from exploiting spatial correlations. A promising avenue for future work is to train models on multi-pixel inputs so that spatial context can improve robustness to anatomical variation and mitigate noise. Generating such datasets, however, is substantially more demanding because variability across numerous organs and tissues must be modelled with higher fidelity.

Overall, this work underscores the importance of variability-aware synthetic datasets in advancing machine learning models for DECT elemental decomposition. By capturing realistic tissue composition variability, these datasets can improve model generalizability and accuracy for applications ranging from image reconstruction to radiation therapy planning and highlight key areas where further methodological and data developments are required.

## 5 Conclusion

This study demonstrates the feasibility of modelling intra- and inter-patient variability in elemental tissue composition to generate synthetic training data for DECT material decomposition. Both linear regression and neural network models performed well under noise-free conditions, with the linear model achieving slightly lower RMSE values. Under noisy conditions, performance degraded for both models, though the linear model generally retained lower numerical error. The neural network, however, consistently produced non-negative mass fractions and showed a small performance advantage for carbon in bone tissue across noise levels. The authors are not aware of prior studies that systematically incorporate this form of elemental variability into synthetic datasets. Future work should incorporate organ-specific tissue statistics, covariances between elemental mass fractions, and spatial context to further enhance model robustness and clinical relevance. These advancements could substantially improve patient-specific radiotherapy planning and CT-based medical imaging overall.

## Supplementary Material

ncaf179_2025_RPD_OXMI_LM_vs_NN_supplementary_material_2025_11_19a
