# Supplementary material for: Optimizing material composition determination in dual-energy computed tomography: a comparative study of a linear model and a fully connected neural network
Source: Radiat Prot Dosimetry. 2026 Mar 13;202(3-4):172–9. doi: 10.1093/rpd/ncaf179 (PMC13046066; doi:10.1093/rpd/ncaf179)
Supplement: ncaf179_2025_RPD_OXMI_LM_vs_NN_supplementary_material_2025_11_19a [file ncaf179_2025_rpd_oxmi_lm_vs_nn_supplementary_material_2025_11_19a.pdf]

Supplementary Information to "Optimizing Material Composition Determination in Dual-Energy CT: A Comparative Study of a Linear Model and a Fully Connected Neural Network"

Alexandr Malusek[2,3], Sofie Malmodin[1], Maria Magnusson[1,3], Michael Sandborg[2,3,4], and Åsa Carlsson Tedgren[2,3,5]

[1]Department of Electrical Engineering, Linköping University, Linköping, Sweden
[2]Department of Health, Medicine and Caring Sciences, Linköping University, Linköping, Sweden
[3]Center for Medical Image Science and Visualization (CMIV), Linköping University, Linköping, Sweden
[4]Department of Medical Physics, Linköping University Hospital, Linköping, Sweden
[5]Department of Nuclear Medicine and Medical Physics, Karolinska University Hospital, Stockholm, Sweden

November 2025

Contents

1 Neural Network **2**
1.1 Architecture . . . 2
1.2 Training Behavior and Performance . . . 2
1.2.1 Training Convergence . . . 2
1.2.2 Generalization Gap . . . 2
1.2.3 Prediction vs. Ground Truth . . . 3
1.2.4 Network Weights and Biases . . . 3
1.3 Extended Simulation with 100,000 Samples . . . 14
1.3.1 Training Convergence . . . 14
1.3.2 Generalization Gap . . . 14
1.3.3 Prediction vs. Ground Truth . . . 14
1.3.4 Network Weights and Biases . . . 14

2 Linear Model **25**
2.1 Parameters . . . 25

This document provides supplementary information supporting the article "Optimizing Material Composition Determination in Dual-Energy CT: A Comparative Study of a Linear Model and a Fully Connected Neural Network". It includes additional methods, figures, tables, and implementation details.

1 Neural Network

1.1 Architecture

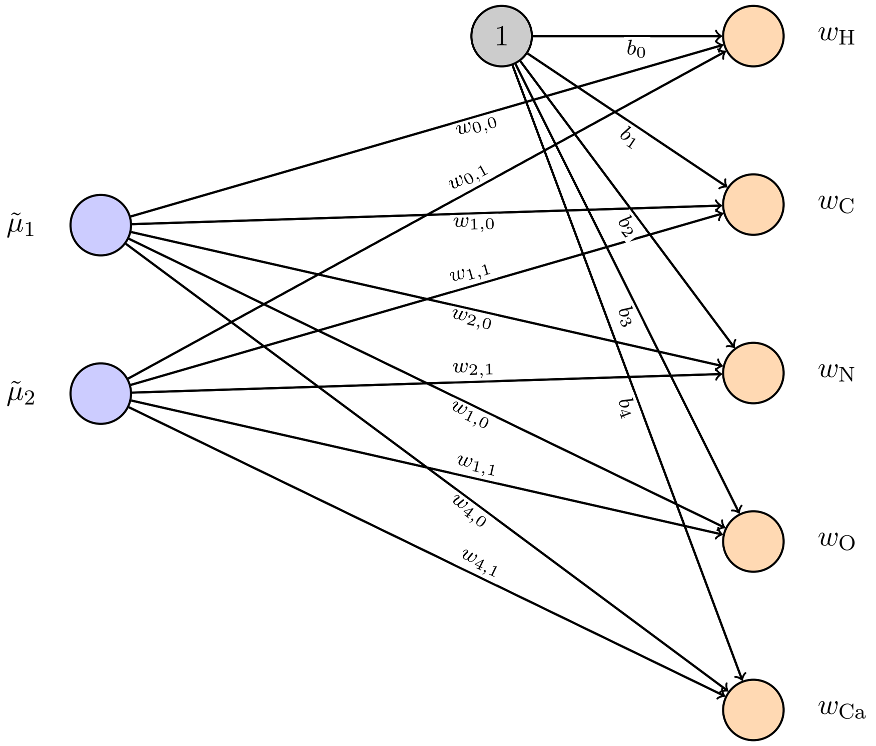

Figure 1: Schematic illustration of a fully connected neural network with two input nodes representing the standardized linear attenuation coefficients ($\tilde{\mu}_1$ and $\tilde{\mu}_2$) and five output nodes corresponding to the elemental mass fractions (w_{H}, w_{C}, w_{N}, w_{O}, and w_{Ca}). The network has no hidden layer. Weights and biases follow the PyTorch tensor indexing convention, where the first index refers to the output neuron and the second to the input neuron. The network ends with a five-dimensional softmax layer (not shown), ensuring that predicted mass fractions sum to unity.

1.2 Training Behavior and Performance

1.2.1 Training Convergence

Figure 2 presents the training and validation loss as functions of the epoch number. For all noise levels, the two curves converge and visually overlap from approximately epoch 40 onward, after which both losses remain nearly constant. Although the losses appear flat on the large dynamic y-axis range, small residual variations are present; these are examined in more detail in Section 1.3.1. As expected, the plateau value increases with higher simulated noise (i.e., lower signal-to-noise ratio, SNR). Simulated noise was added to the calculated linear attenuation coefficients.

The uncertainty bands show that repeated training runs yield highly consistent loss values, indicating stable optimization behavior. The MSE-based loss is systematically lower for bone tissue than for soft tissue, consistent with the prediction-versus-ground-truth results shown in Figures 4—9.

1.2.2 Generalization Gap

Figure 3 shows the generalization gap, defined as the difference between the validation and training loss, as a function of the epoch number. A small gap indicates good generalization, meaning that the model performs similarly on unseen data and on the training set. For all SNR levels, the gap decreases rapidly during the first few epochs and then stabilizes at low values, consistent with the convergence behavior shown in Figure 2. No positive generalization gap—typically associated with overfitting—was observed.

1.2.3 Prediction vs. Ground Truth

Figures 4–9 show the predicted values versus the ground-truth values for soft and bone tissues across the three noise levels. For each tissue–noise combination, ten independently trained models were evaluated. While the detailed interpretation of these results is provided in the main article, the plots shown here demonstrate that the variability between individual model realizations is small. Although the weights and biases differ between models (Tables 1 and 2), their predictive performance is highly consistent.

1.2.4 Network Weights and Biases

The neural network weights and biases are listed in Tables 1 and 2.

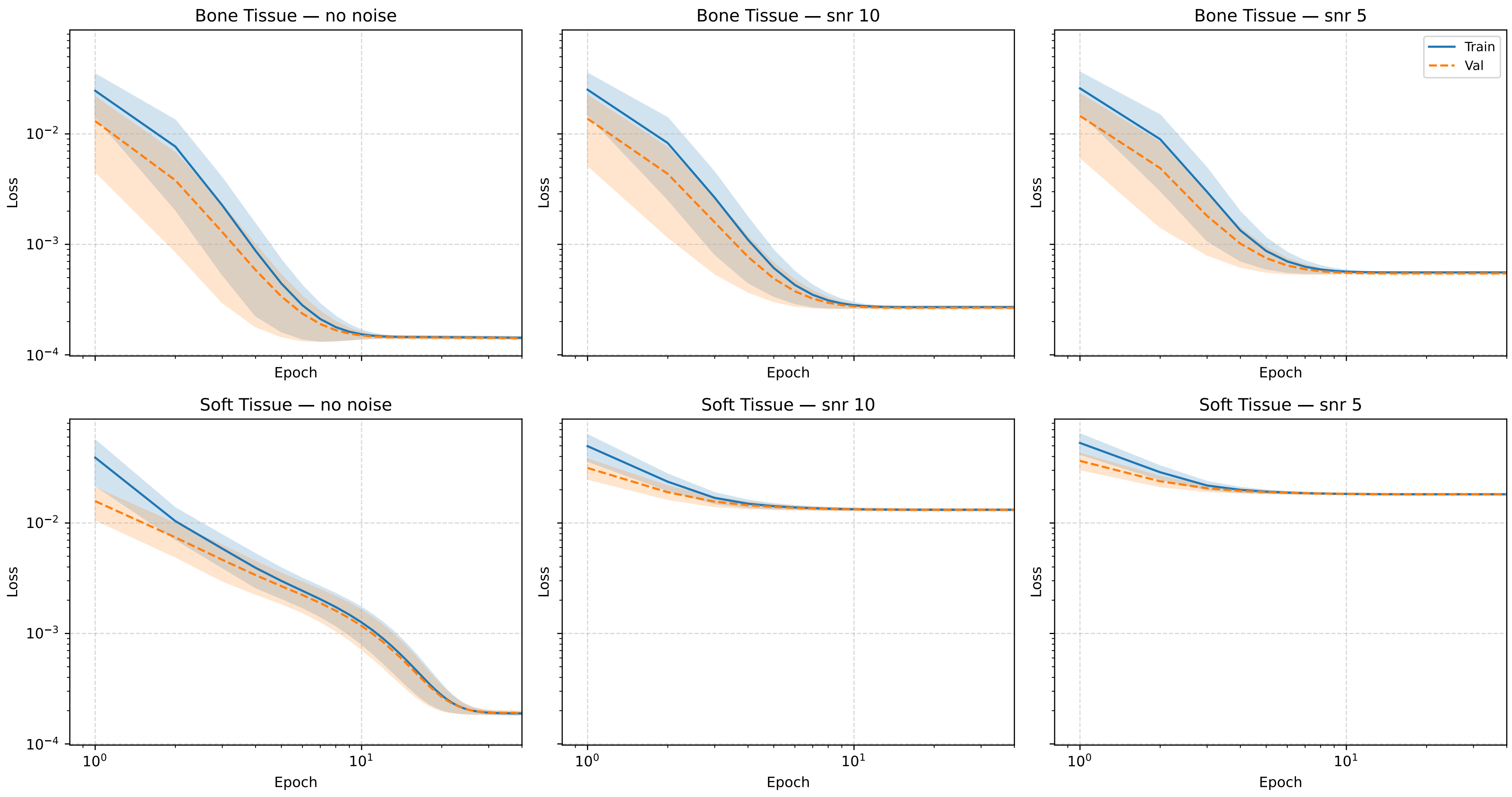

Figure 2: Training and validation loss as functions of the number of epochs for bone and soft tissues, evaluated at noise levels of no noise, SNR = 10, and SNR = 5, using a dataset of 10,000 samples. Shaded areas around the curves represent the standard uncertainties.

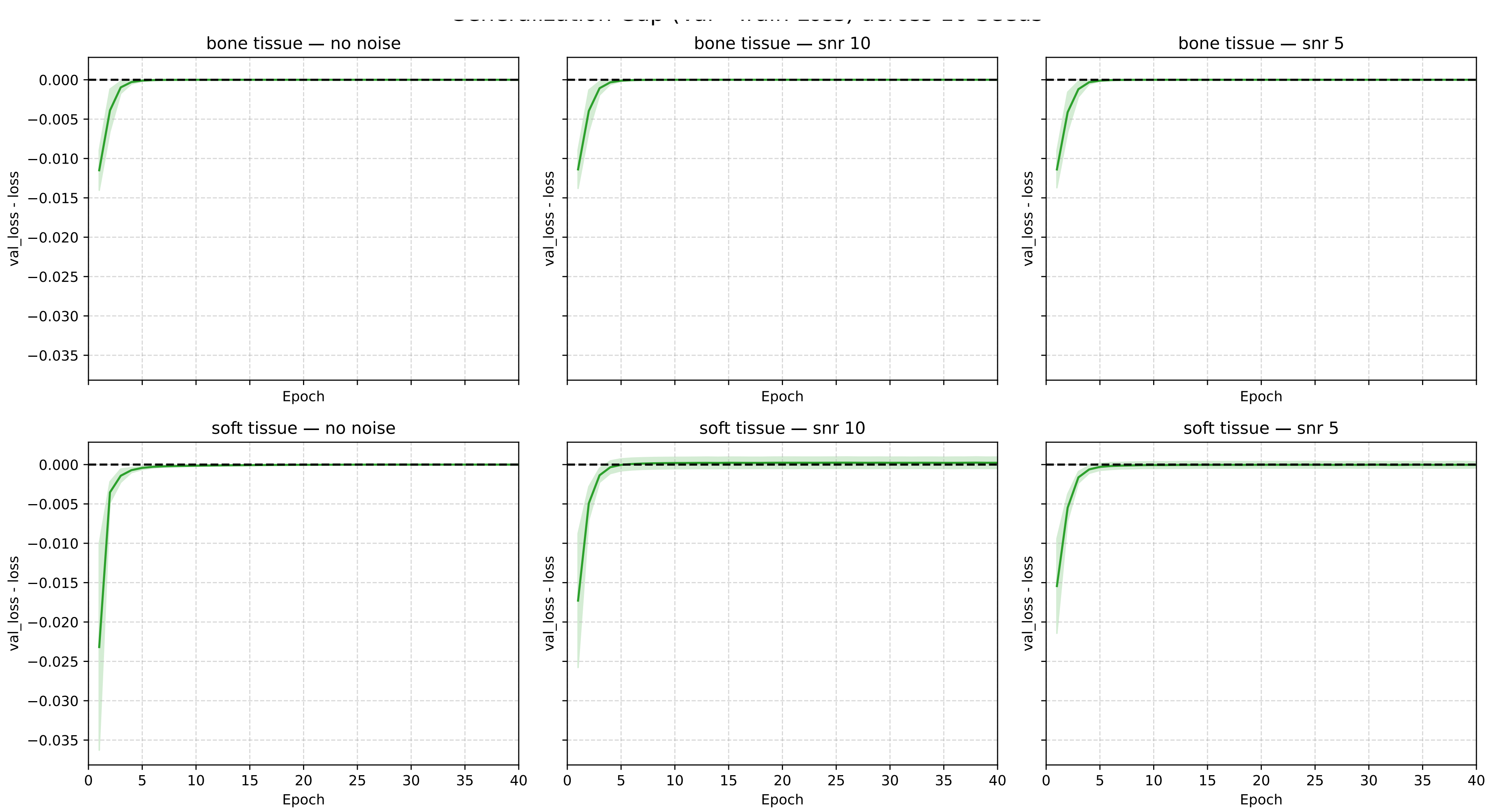

Figure 3: Difference between validation and training loss as a function of the number of epochs for bone and soft tissues, evaluated at noise levels of no noise, SNR = 10, and SNR = 5, using a dataset of 10,000 samples. Shaded regions around the curves indicate the standard uncertainties.

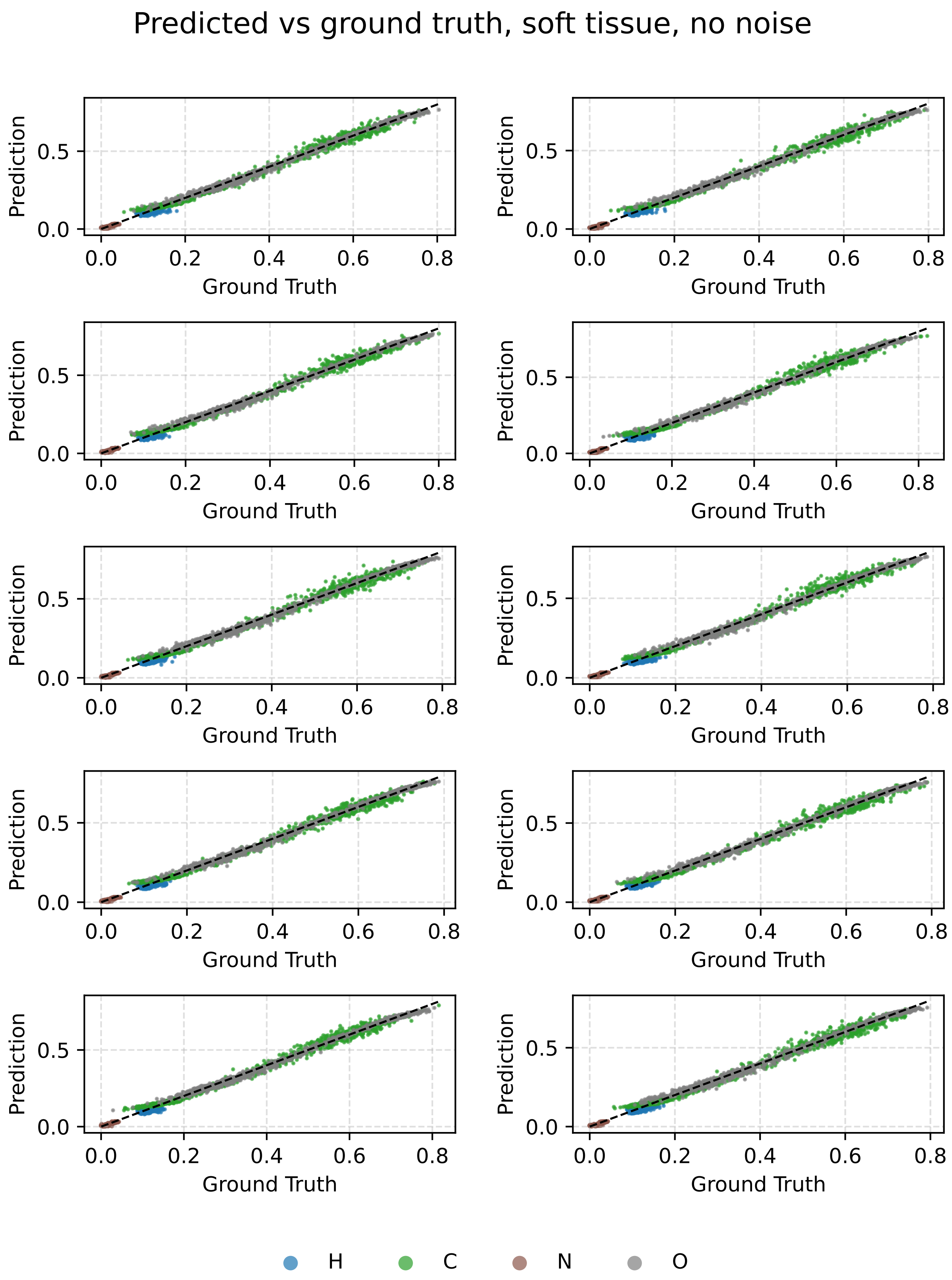

Figure 4: Predictions of the neural network versus ground truth for soft tissues under noise-free conditions, using a dataset of 10,000 samples. Only 500 randomly selected points are shown. Each of the ten panels represents an independent simulation.

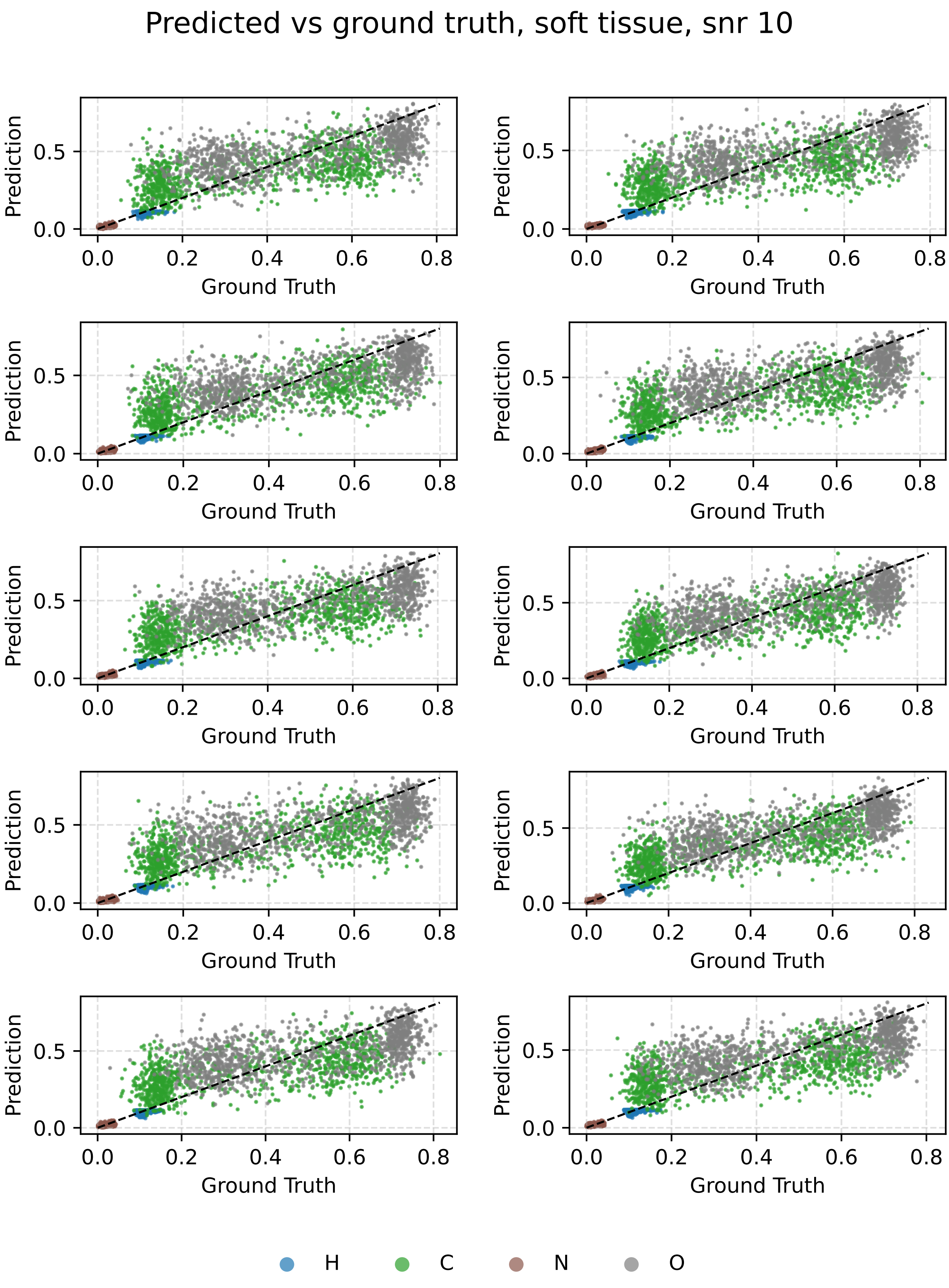

Figure 5: Same as Figure 4, but for a signal-to-noise ratio of 10.

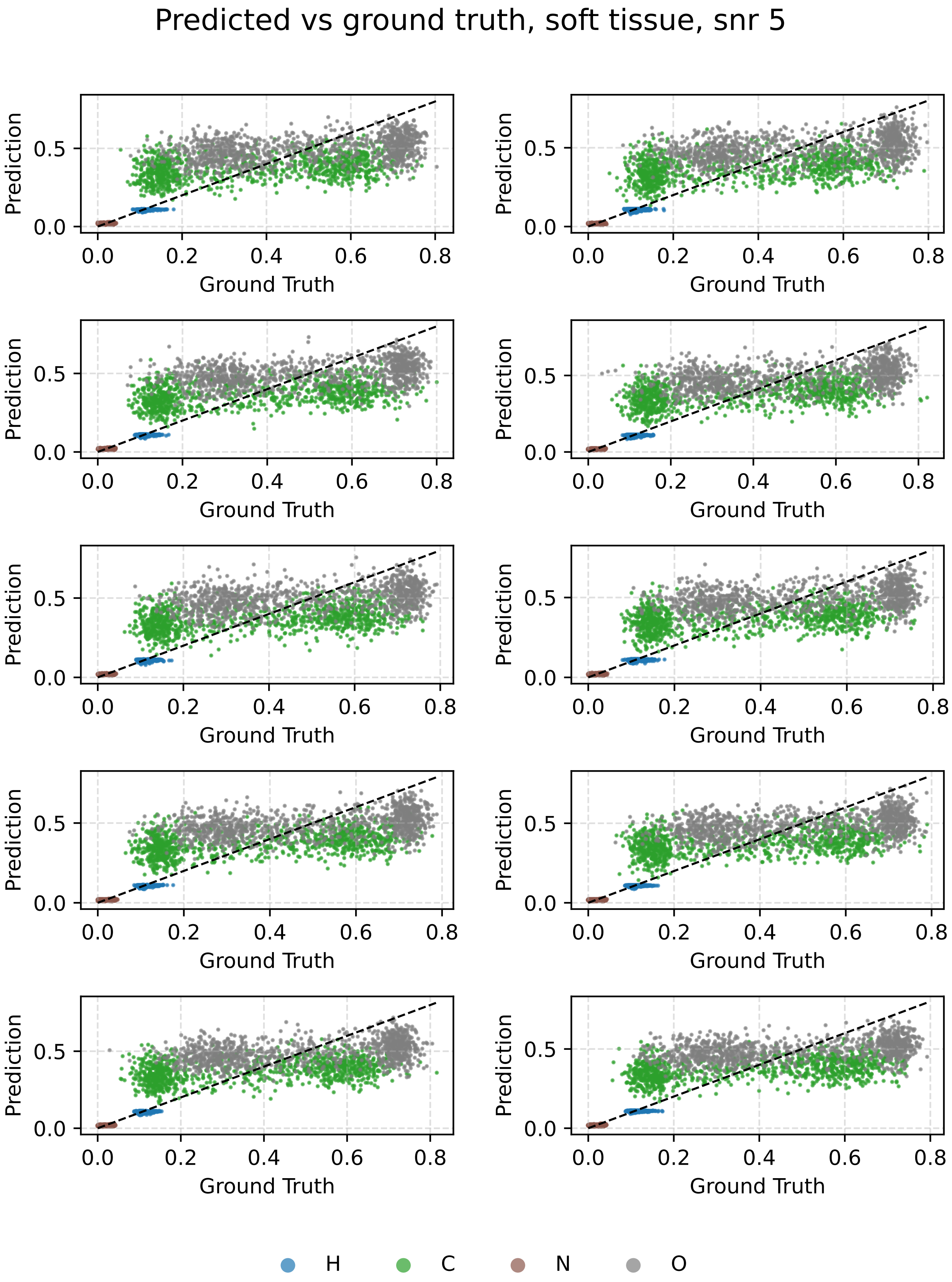

Figure 6: Same as Figure 4, but for a signal-to-noise ratio of 5.

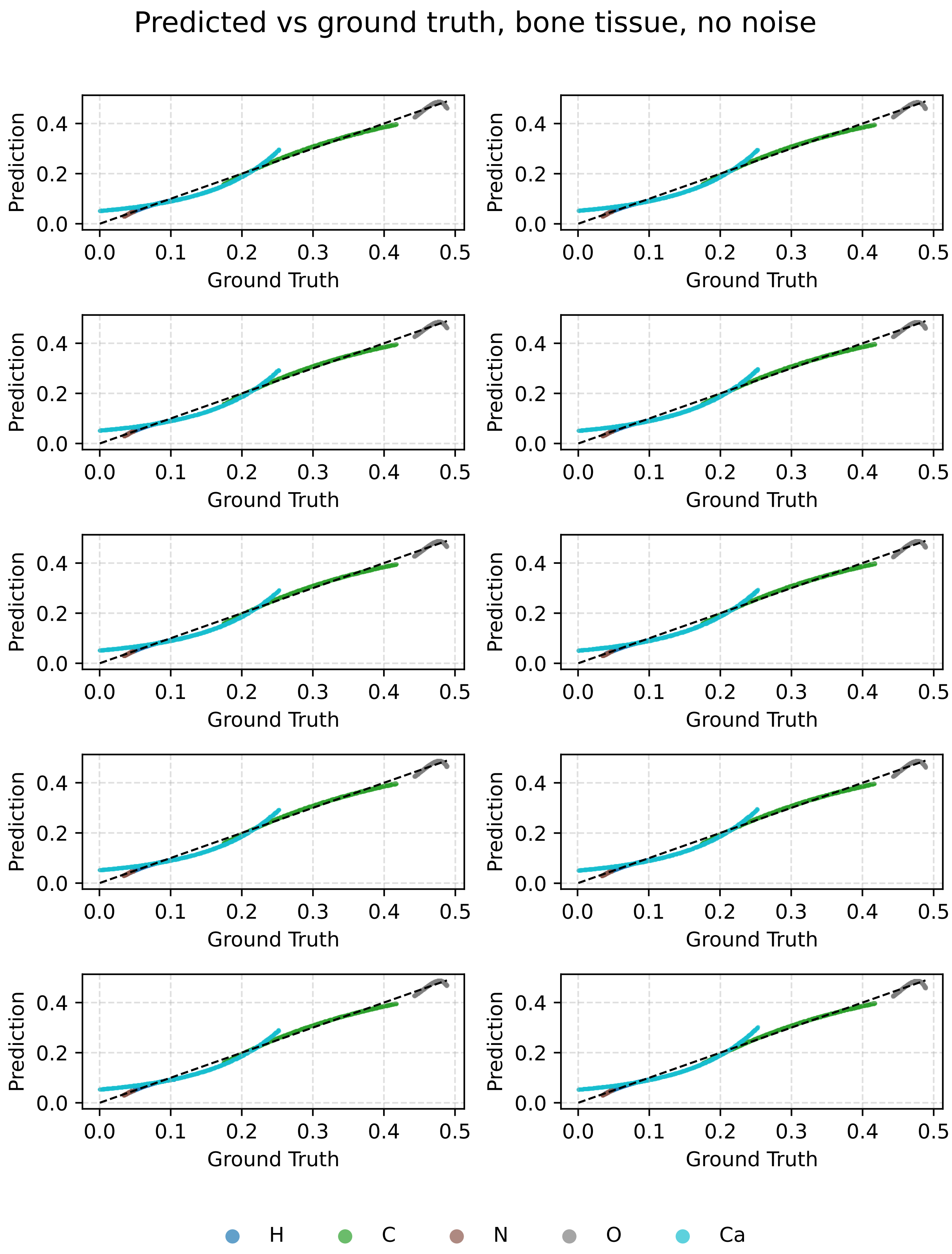

Figure 7: Predictions of the neural network versus ground truth for bone tissue under noise-free conditions, using a dataset of 10,000 samples. Each of the ten panels represents an independent simulation.

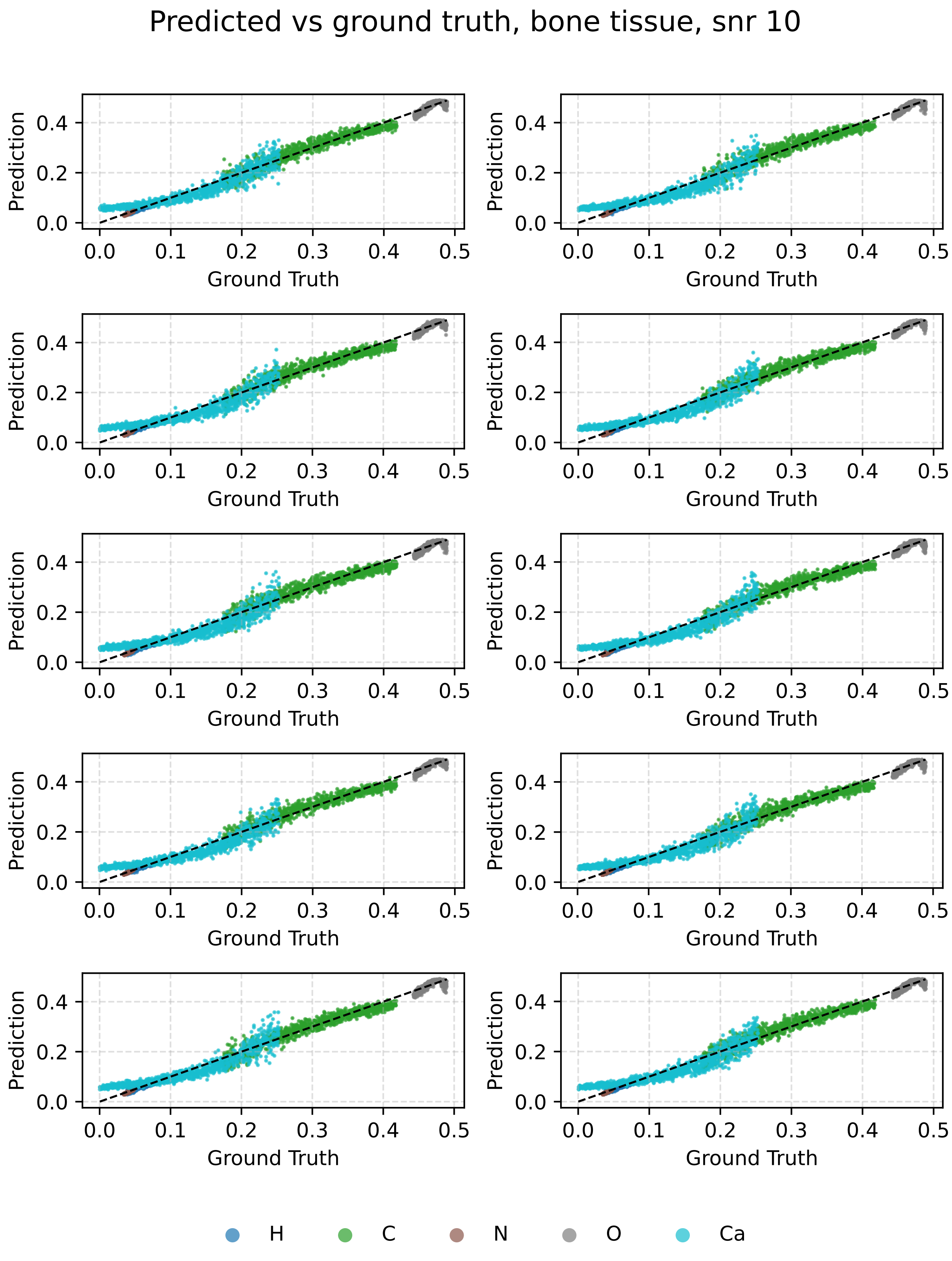

Figure 8: Same as Figure 7, but for a signal-to-noise ratio of 10.

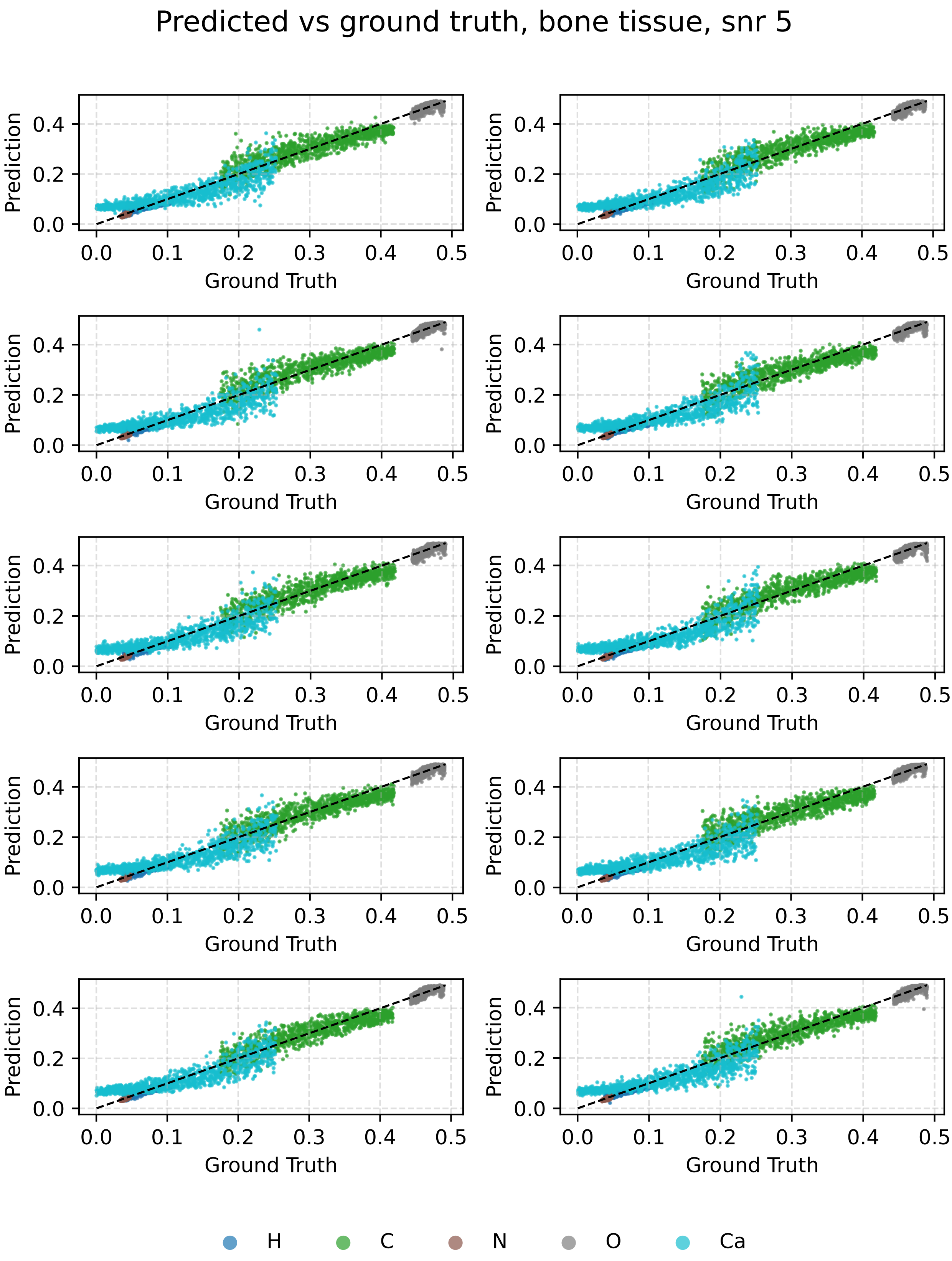

Figure 9: Same as Figure 7, but for a signal-to-noise ratio of 5.

Table 1: Weights and biases of the neural network applied to bone tissue data. The network architecture and parameter notation are illustrated in Figure 1.

		H		C		N		O		Ca		H	C	N	O	Ca
SNR	i	$w_{0,0}$	$w_{0,1}$	$w_{1,0}$	$w_{1,1}$	$w_{2,0}$	$w_{2,1}$	$w_{3,0}$	$w_{3,1}$	$w_{4,0}$	$w_{4,1}$	b_0	b_1	b_2	b_3	b_4
∞	0	-0.581	0.383	0.220	-0.410	-0.515	0.715	0.038	0.054	-0.174	0.733	-1.362	0.058	-1.946	0.525	-0.920
∞	1	-0.055	-0.068	0.077	-0.207	-0.766	1.027	0.095	0.056	0.066	0.546	-0.772	0.639	-1.350	1.105	-0.329
∞	2	-0.371	-0.080	0.268	-0.724	-0.309	0.245	0.420	-0.595	0.223	0.064	-0.584	0.828	-1.164	1.295	-0.143
∞	3	-0.598	0.105	0.321	-0.814	-0.157	0.049	0.188	-0.400	-0.409	0.667	-1.077	0.338	-1.649	0.804	-0.632
∞	4	-0.749	-0.154	-0.175	-0.719	-0.592	0.077	0.008	-0.622	-0.652	0.496	-0.408	1.008	-0.985	1.474	0.034
∞	5	0.255	-0.057	0.480	-0.284	0.453	0.131	0.127	0.352	-0.519	1.460	-0.864	0.551	-1.445	1.018	-0.421
∞	6	-0.353	0.280	0.567	-0.632	-0.325	0.641	0.339	-0.123	0.300	0.374	-0.491	0.929	-1.064	1.394	-0.043
∞	7	-0.172	-0.054	0.230	-0.455	-0.424	0.585	0.007	0.051	-0.419	0.942	-0.890	0.524	-1.470	0.991	-0.450
∞	8	-0.445	0.332	0.796	-0.900	-0.309	0.582	0.965	-0.788	0.327	0.301	-0.887	0.538	-1.457	1.004	-0.431
∞	9	-0.007	0.107	0.186	-0.087	-0.097	0.593	-0.026	0.408	0.102	0.750	-0.832	0.582	-1.411	1.052	-0.380
10	0	-0.295	0.093	-0.305	0.097	-0.006	0.207	-0.093	0.180	0.211	0.331	-1.365	0.056	-1.949	0.520	-0.903
10	1	-0.292	0.170	-0.302	0.172	-0.006	0.282	-0.097	0.251	0.190	0.411	-0.770	0.645	-1.350	1.108	-0.310
10	2	-0.058	-0.379	-0.068	-0.377	0.221	-0.261	0.136	-0.297	0.416	-0.137	-0.586	0.826	-1.171	1.287	-0.139
10	3	-0.300	-0.185	-0.309	-0.179	-0.021	-0.071	-0.109	-0.094	0.183	0.064	-1.084	0.330	-1.665	0.795	-0.631
10	4	-0.517	-0.330	-0.520	-0.327	-0.241	-0.217	-0.317	-0.246	-0.025	-0.100	-0.419	0.994	-1.002	1.456	0.029
10	5	-0.157	0.284	-0.165	0.287	0.127	0.395	0.041	0.366	0.346	0.516	-0.842	0.573	-1.423	1.039	-0.384
10	6	0.037	-0.091	0.032	-0.089	0.316	0.020	0.237	-0.007	0.529	0.147	-0.498	0.916	-1.074	1.380	-0.047
10	7	-0.223	-0.023	-0.229	-0.023	0.054	0.099	-0.026	0.063	0.256	0.233	-0.894	0.518	-1.473	0.985	-0.431
10	8	0.180	-0.237	0.173	-0.227	0.460	-0.123	0.378	-0.144	0.667	0.023	-0.881	0.536	-1.454	1.006	-0.411
10	9	-0.128	0.231	-0.134	0.232	0.150	0.349	0.071	0.315	0.361	0.470	-0.825	0.593	-1.406	1.056	-0.363
5	0	-0.266	0.091	-0.277	0.090	-0.001	0.224	-0.085	0.182	0.175	0.342	-1.357	0.059	-1.944	0.520	-0.883
5	1	-0.268	0.148	-0.280	0.150	0.002	0.273	-0.083	0.239	0.179	0.391	-0.768	0.648	-1.351	1.112	-0.295
5	2	-0.053	-0.365	-0.060	-0.362	0.215	-0.240	0.131	-0.279	0.394	-0.128	-0.597	0.816	-1.187	1.275	-0.132
5	3	-0.286	-0.173	-0.294	-0.170	-0.023	-0.045	-0.105	-0.080	0.155	0.075	-1.090	0.320	-1.669	0.782	-0.622
5	4	-0.468	-0.302	-0.477	-0.298	-0.197	-0.171	-0.283	-0.204	-0.022	-0.043	-0.443	0.973	-1.031	1.432	0.034
5	5	-0.190	0.182	-0.201	0.184	0.097	0.305	0.000	0.269	0.279	0.407	-0.828	0.585	-1.412	1.047	-0.347
5	6	0.063	-0.073	0.053	-0.073	0.334	0.052	0.248	0.014	0.509	0.159	-0.518	0.892	-1.096	1.356	-0.049
5	7	-0.234	-0.043	-0.240	-0.043	0.031	0.080	-0.050	0.043	0.211	0.190	-0.889	0.521	-1.475	0.988	-0.422
5	8	0.220	-0.185	0.216	-0.182	0.487	-0.063	0.409	-0.096	0.682	0.054	-0.874	0.540	-1.455	1.006	-0.397
5	9	-0.116	0.210	-0.130	0.208	0.145	0.338	0.062	0.297	0.319	0.453	-0.814	0.602	-1.395	1.059	-0.340

Table 2: Weights and biases of the neural network for soft tissues.

		H		C		N		O		H	C	N	O
SNR	i	$w_{0,0}$	$w_{0,1}$	$w_{1,0}$	$w_{1,1}$	$w_{2,0}$	$w_{2,1}$	$w_{3,0}$	$w_{3,1}$	b_0	b_1	b_2	b_3
∞	0	-0.724	0.803	-1.834	1.362	0.719	-0.038	1.376	-0.833	-0.482	0.579	-2.267	0.951
∞	1	-0.544	0.675	-1.652	1.251	0.754	0.004	1.555	-0.959	-0.090	0.963	-1.881	1.341
∞	2	-0.520	0.202	-1.609	0.745	0.835	-0.537	1.584	-1.438	-0.255	0.804	-2.044	1.174
∞	3	-0.629	-0.056	-1.691	0.460	0.883	-0.954	1.511	-1.735	-0.076	0.995	-1.867	1.362
∞	4	-0.666	0.442	-1.735	0.950	0.728	-0.353	1.488	-1.260	-0.469	0.585	-2.254	0.958
∞	5	-0.262	0.801	-1.331	1.310	1.330	-0.192	1.858	-0.869	-0.256	0.815	-2.038	1.179
∞	6	-0.026	0.433	-1.089	0.961	1.213	-0.191	2.133	-1.247	-0.418	0.640	-2.214	1.008
∞	7	-0.464	0.569	-1.528	1.090	0.831	-0.106	1.675	-1.105	0.111	1.164	-1.679	1.538
∞	8	0.013	0.055	-1.094	0.607	1.240	-0.554	2.129	-1.605	0.145	1.220	-1.642	1.584
∞	9	-0.730	1.140	-1.775	1.653	0.739	0.288	1.416	-0.540	0.254	1.312	-1.537	1.688
10	0	-0.236	0.148	-0.468	-0.021	0.114	0.376	0.013	0.296	-0.681	0.484	-2.421	0.798
10	1	-0.213	0.181	-0.434	0.038	0.106	0.387	0.032	0.339	-0.360	0.816	-2.090	1.135
10	2	0.016	-0.261	-0.233	-0.432	0.382	-0.018	0.261	-0.112	-0.493	0.677	-2.243	0.986
10	3	-0.241	-0.311	-0.472	-0.456	0.122	-0.061	0.005	-0.147	-0.295	0.878	-2.050	1.199
10	4	-0.046	-0.044	-0.287	-0.202	0.313	0.192	0.198	0.112	-0.754	0.416	-2.513	0.740
10	5	0.112	0.149	-0.123	-0.012	0.481	0.426	0.350	0.322	-0.343	0.825	-2.117	1.136
10	6	0.239	0.120	0.002	-0.042	0.634	0.367	0.504	0.292	-0.609	0.562	-2.379	0.864
10	7	-0.111	0.108	-0.357	-0.034	0.265	0.331	0.156	0.252	-0.162	1.004	-1.916	1.321
10	8	0.320	-0.320	0.089	-0.472	0.700	-0.064	0.578	-0.160	-0.182	0.994	-1.943	1.313
10	9	0.002	0.381	-0.221	0.230	0.321	0.607	0.244	0.528	-0.077	1.087	-1.803	1.409
5	0	-0.204	0.111	-0.348	0.005	-0.083	0.224	-0.064	0.208	-0.724	0.484	-2.378	0.775
5	1	-0.200	0.169	-0.336	0.042	-0.066	0.293	-0.043	0.293	-0.394	0.816	-2.057	1.119
5	2	0.059	-0.218	-0.080	-0.326	0.183	-0.101	0.198	-0.118	-0.535	0.663	-2.182	0.969
5	3	-0.206	-0.247	-0.337	-0.349	-0.063	-0.159	-0.042	-0.150	-0.339	0.883	-2.002	1.174
5	4	-0.028	-0.017	-0.168	-0.113	0.092	0.084	0.137	0.064	-0.785	0.413	-2.440	0.730
5	5	0.108	0.117	-0.019	0.015	0.253	0.238	0.260	0.207	-0.383	0.816	-2.043	1.122
5	6	0.203	0.124	0.072	0.029	0.337	0.228	0.360	0.217	-0.650	0.561	-2.316	0.835
5	7	-0.119	0.096	-0.258	-0.004	0.016	0.186	0.017	0.174	-0.203	1.000	-1.864	1.289
5	8	0.318	-0.261	0.184	-0.351	0.465	-0.156	0.473	-0.183	-0.231	0.982	-1.893	1.277
5	9	0.091	0.390	-0.050	0.293	0.220	0.484	0.219	0.450	-0.124	1.073	-1.770	1.373

1.3 Extended Simulation with 100,000 Samples

The neural network training and testing were repeated using a dataset consisting of 100 000 samples, with 90 000 allocated for training and 10 000 for testing. The number of training epochs was increased from 80 to 500. The results of this extended simulation are presented in this section.

1.3.1 Training Convergence

Figure 10 shows the training and validation loss as a function of epoch number, with the x-axis plotted on a logarithmic scale. Compared with the experiment using 10 000 samples, convergence was noticeably faster: most loss curves reached a plateau after the first epoch. The only exception was the bone-tissue model without added noise, for which a gradual decline in the training loss was observed up to approximately epoch 400, after which the curve stabilized.

1.3.2 Generalization Gap

As shown in Figure 11, the training and validation loss curves began to overlap already after the first epoch. This represents an improvement compared with the simulation using 10 000 samples.

1.3.3 Prediction vs. Ground Truth

Figures 12–17 present the predictions versus ground truth for the dataset containing 90 000 training samples. The scatter plots do not differ noticeably from those obtained using 9 000 training samples, indicating that the smaller dataset provides a representative characterization of the method's performance.

1.3.4 Network Weights and Biases

The neural network weights and biases are listed in Tables 3 and 4.

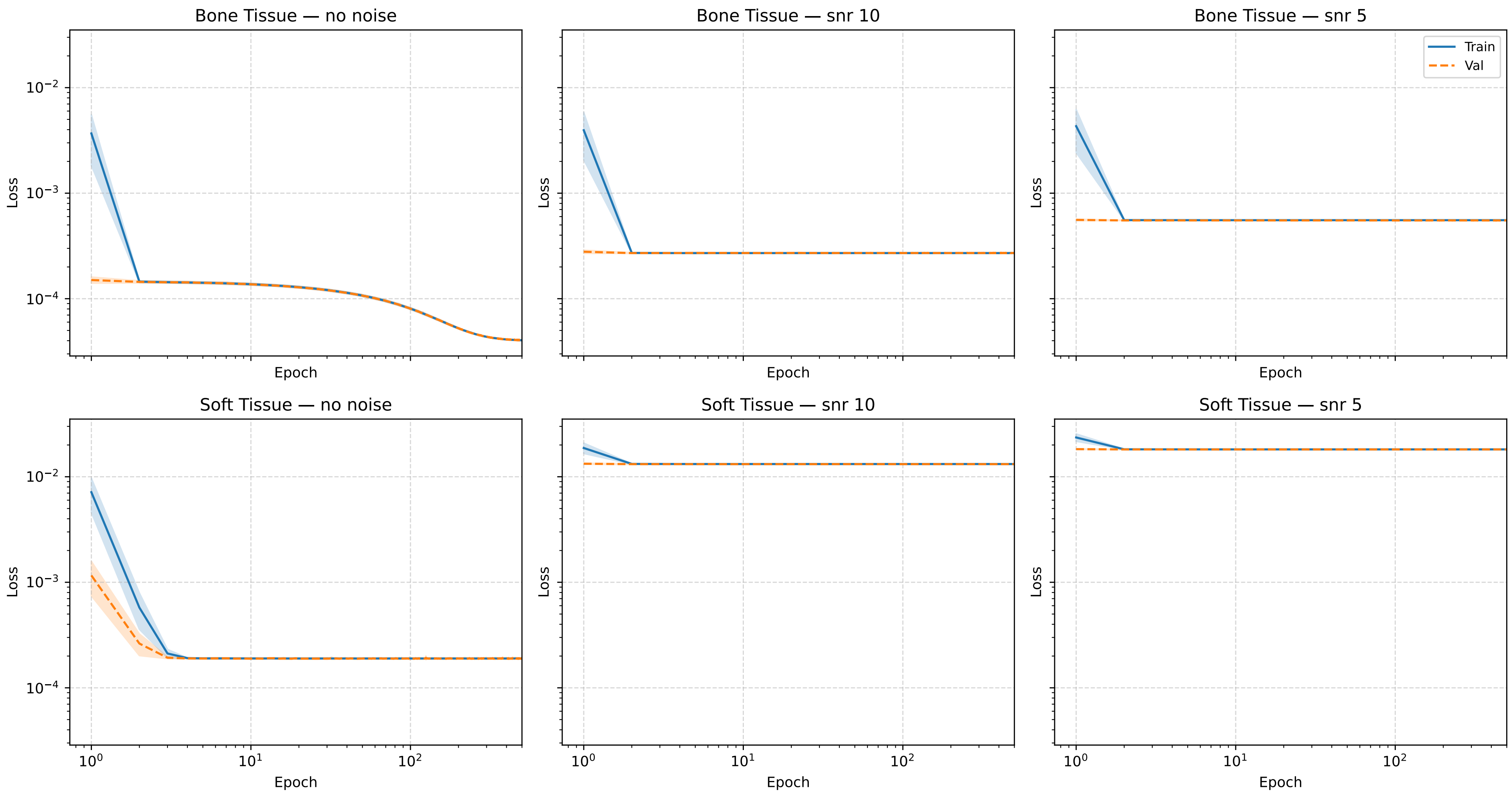

Figure 10: Training and validation loss as functions of the number of epochs for bone and soft tissues, evaluated at noise levels of no noise, SNR = 10, and SNR = 5, using a dataset of 100,000 samples. Shaded areas around the curves represent the standard uncertainties.

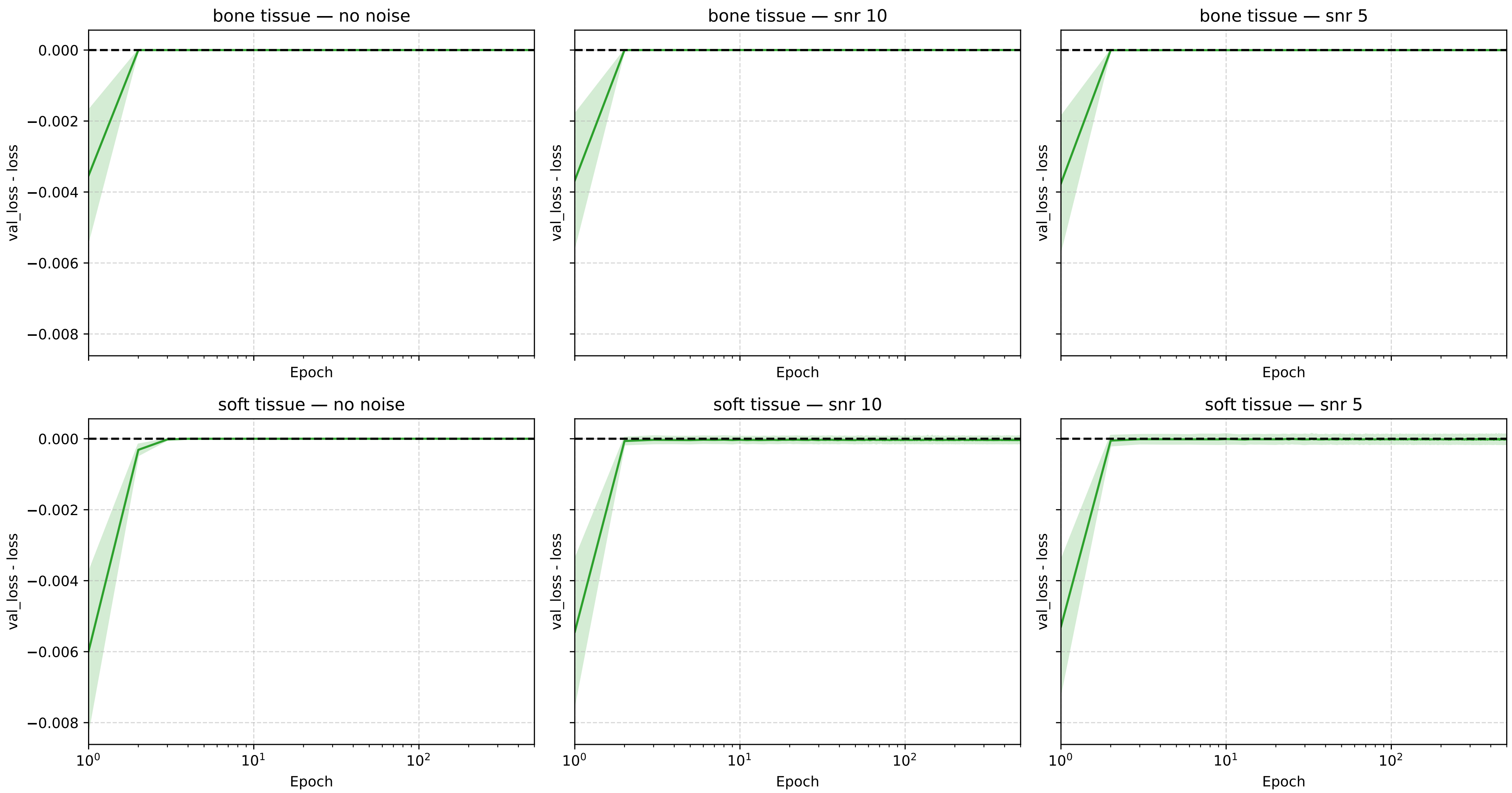

Figure 11: Difference between validation and training loss as a function of the number of epochs for bone and soft tissues, evaluated at noise levels of no noise, SNR = 10, and SNR = 5, using a dataset of 100,000 samples. Shaded regions around the curves indicate the standard uncertainties.

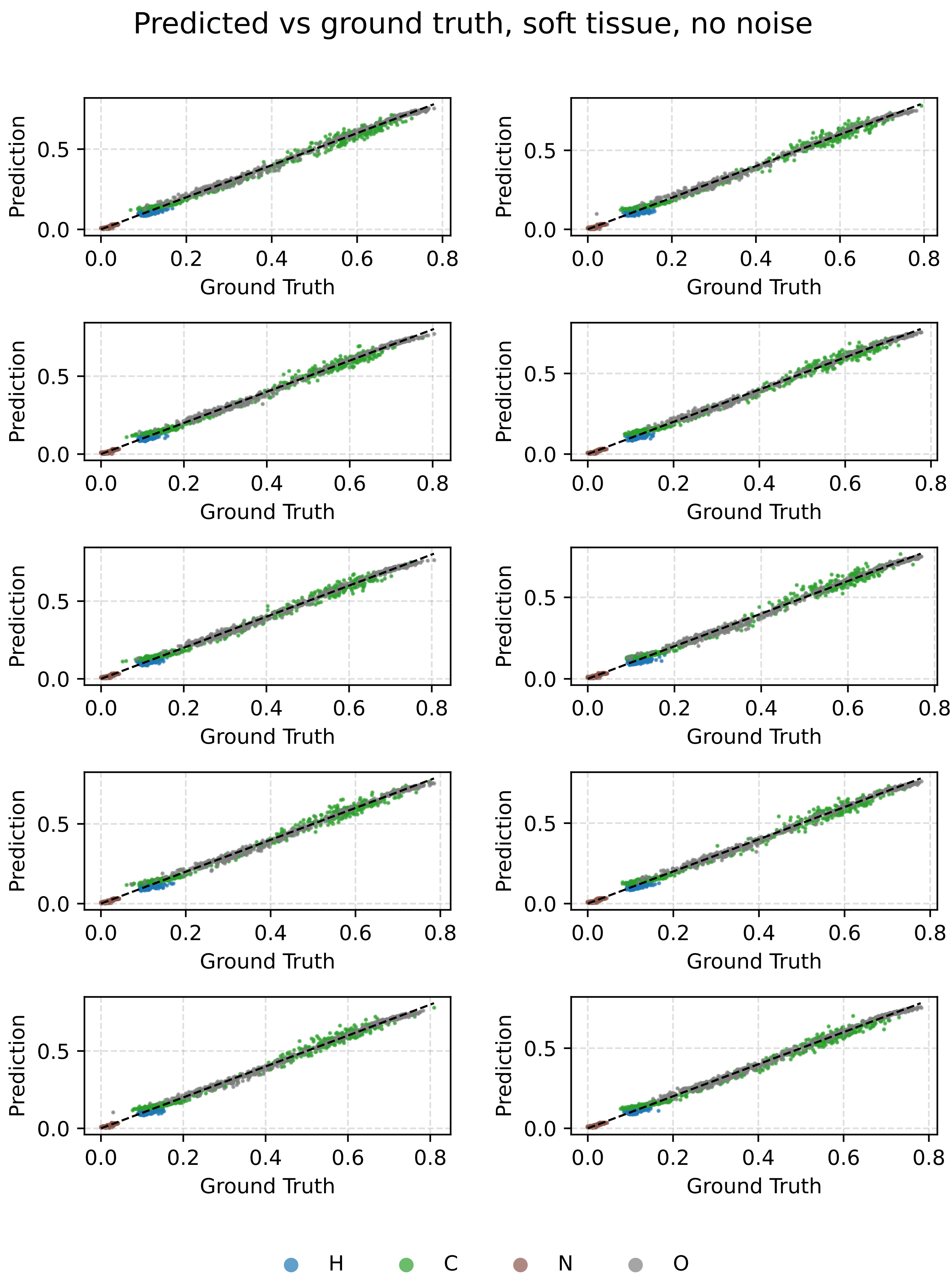

Figure 12: Predictions of the neural network versus ground truth for soft tissues under noise-free conditions, using a dataset of 100,000 samples. Only 500 randomly selected points are shown. Each of the ten panels represents an independent simulation.

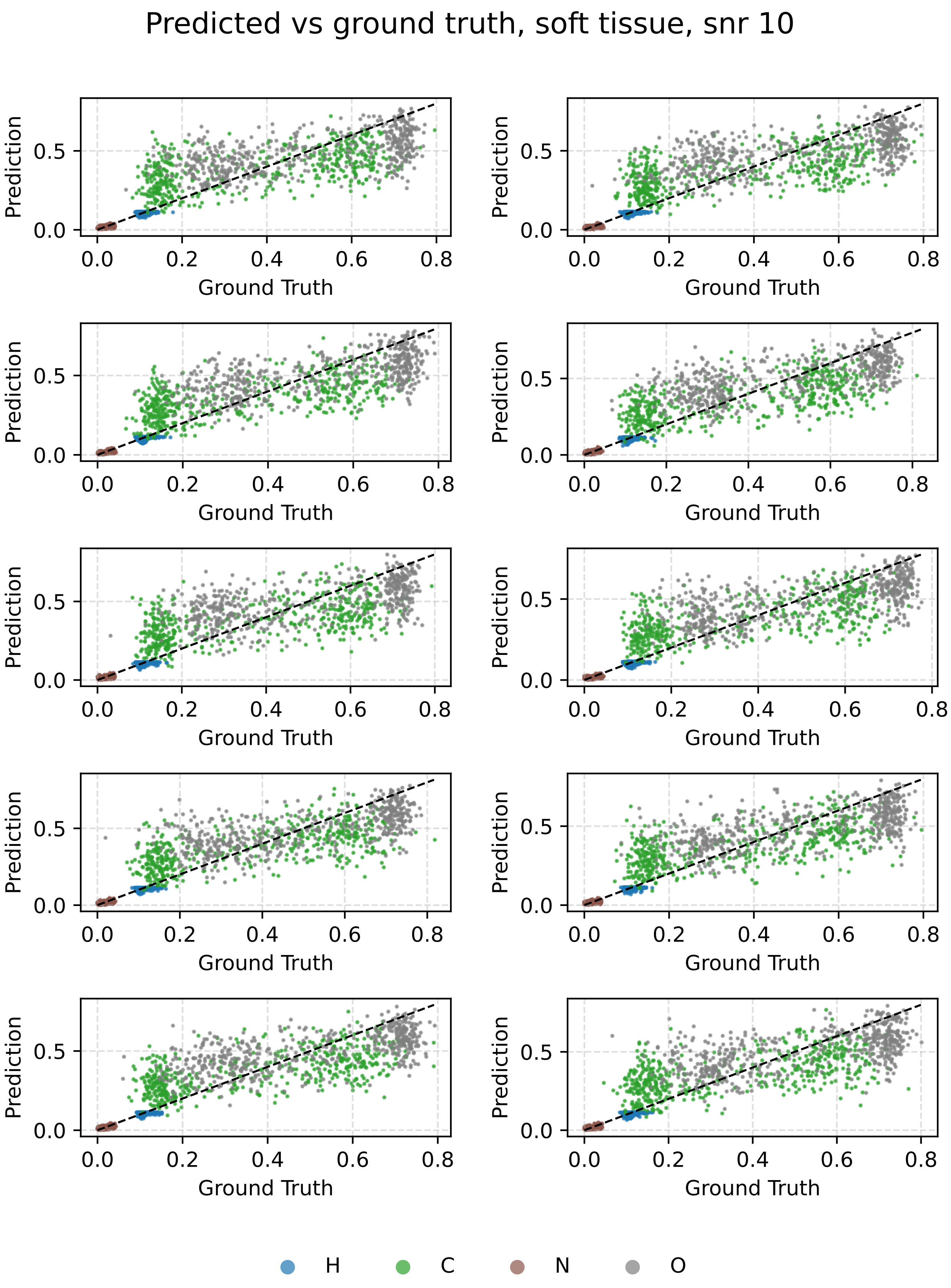

Figure 13: Same as Figure 12, but for a signal-to-noise ratio of 10.

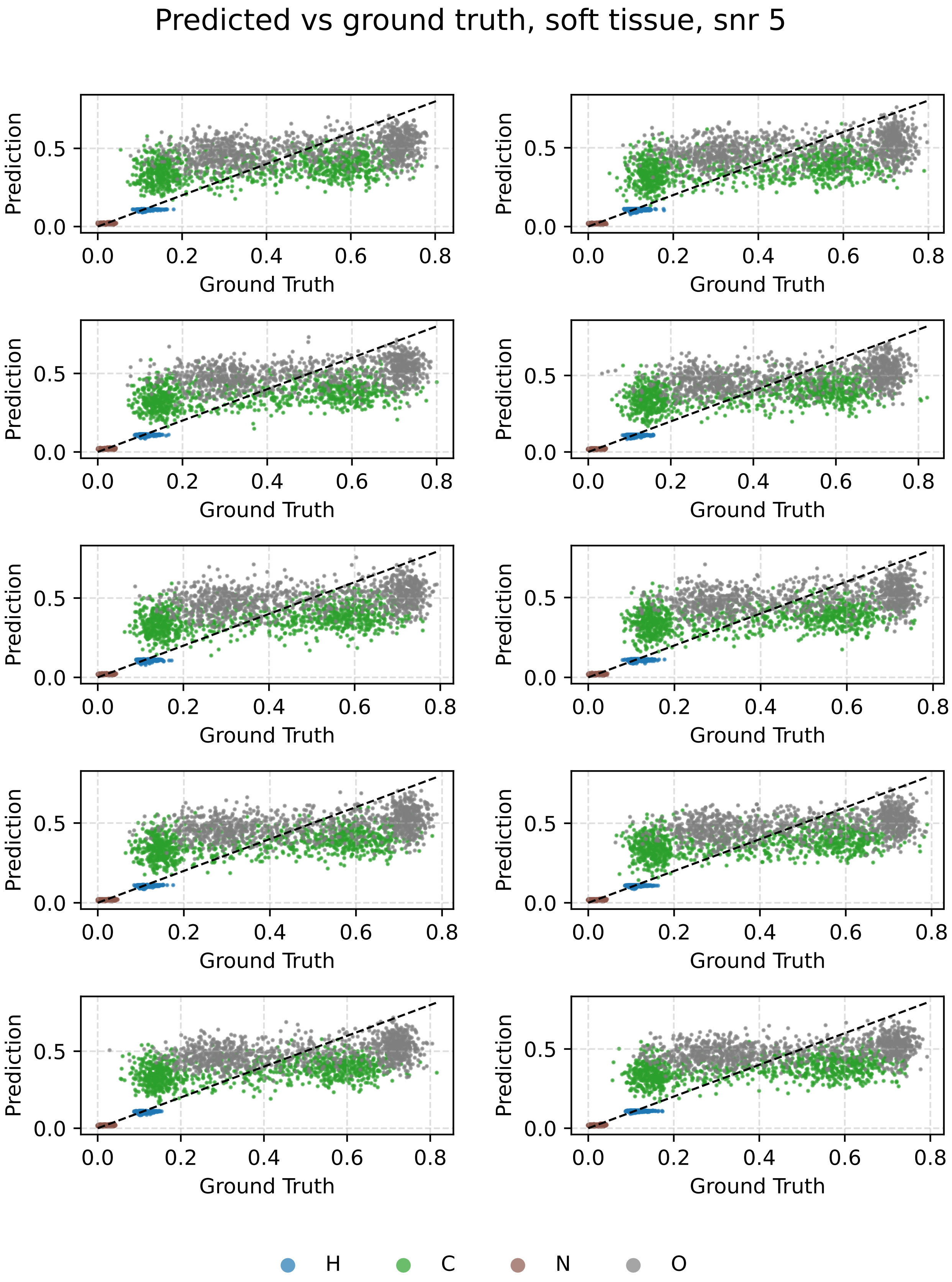

Figure 14: Same as Figure 12, but for a signal-to-noise ratio of 5.

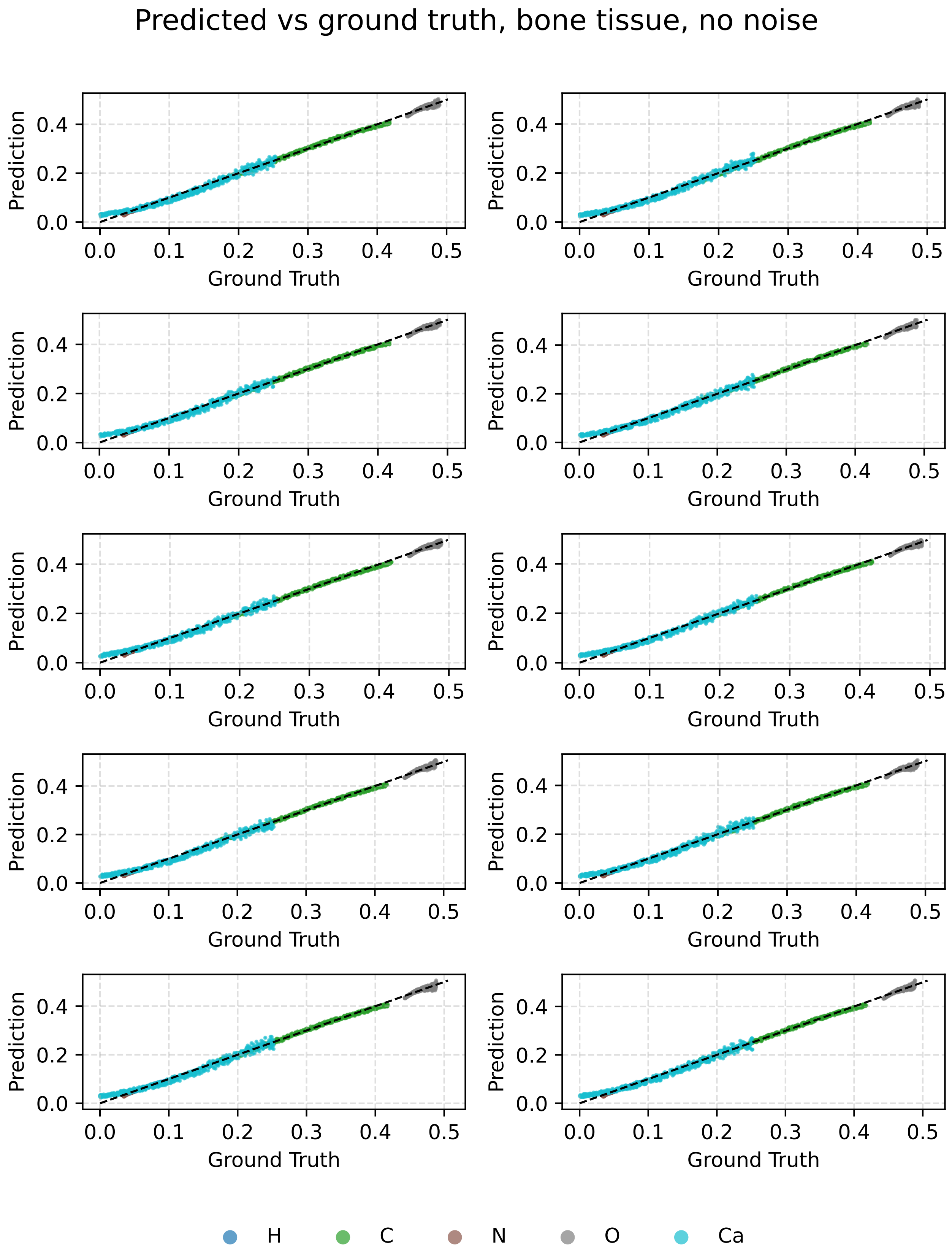

Figure 15: Predictions of the neural network versus ground truth for bone tissue under noise-free conditions, using a dataset of 100,000 samples. Only 500 randomly selected points are shown. Each of the ten panels represents an independent simulation.

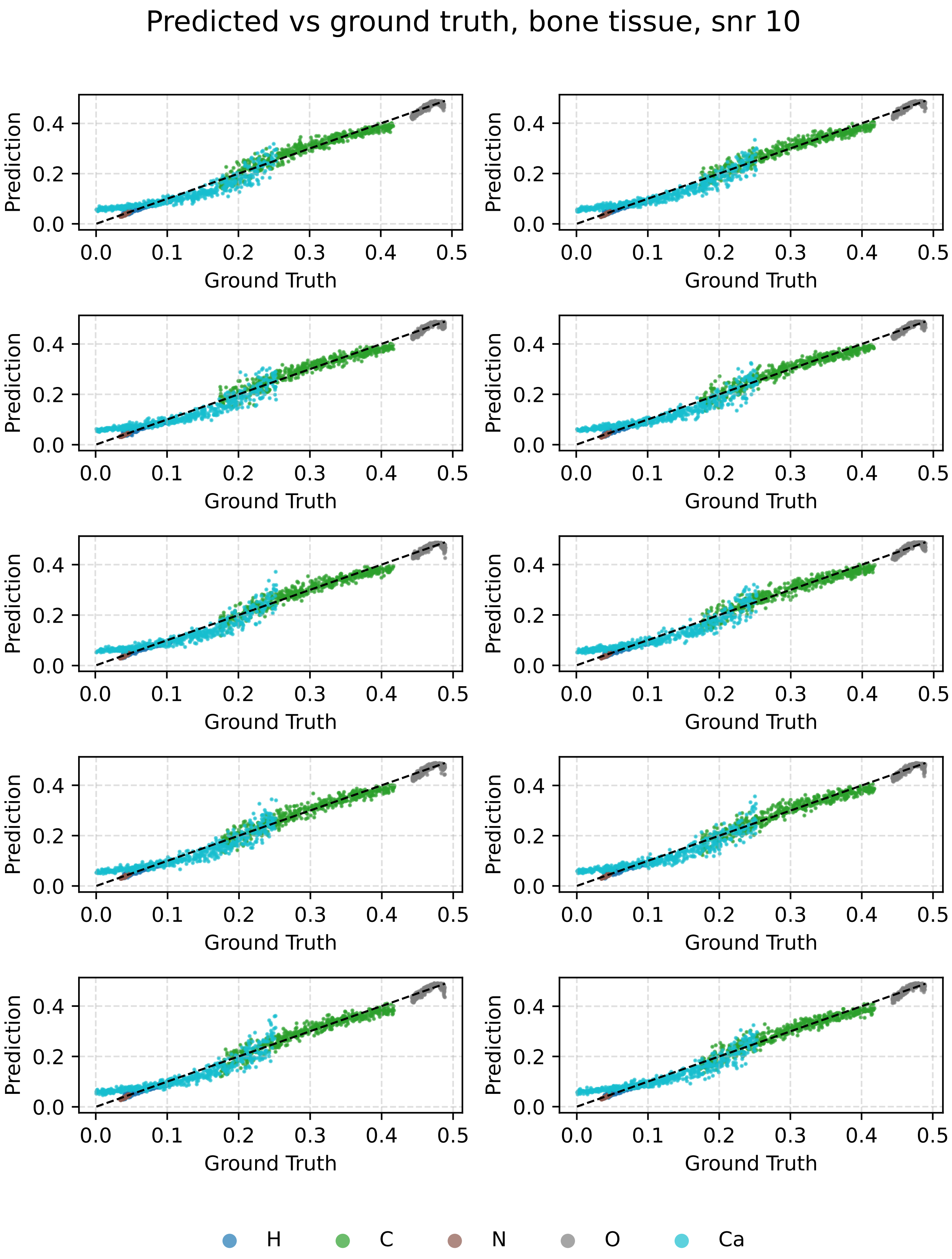

Figure 16: Same as Figure 15, but for a signal-to-noise ratio of 10.

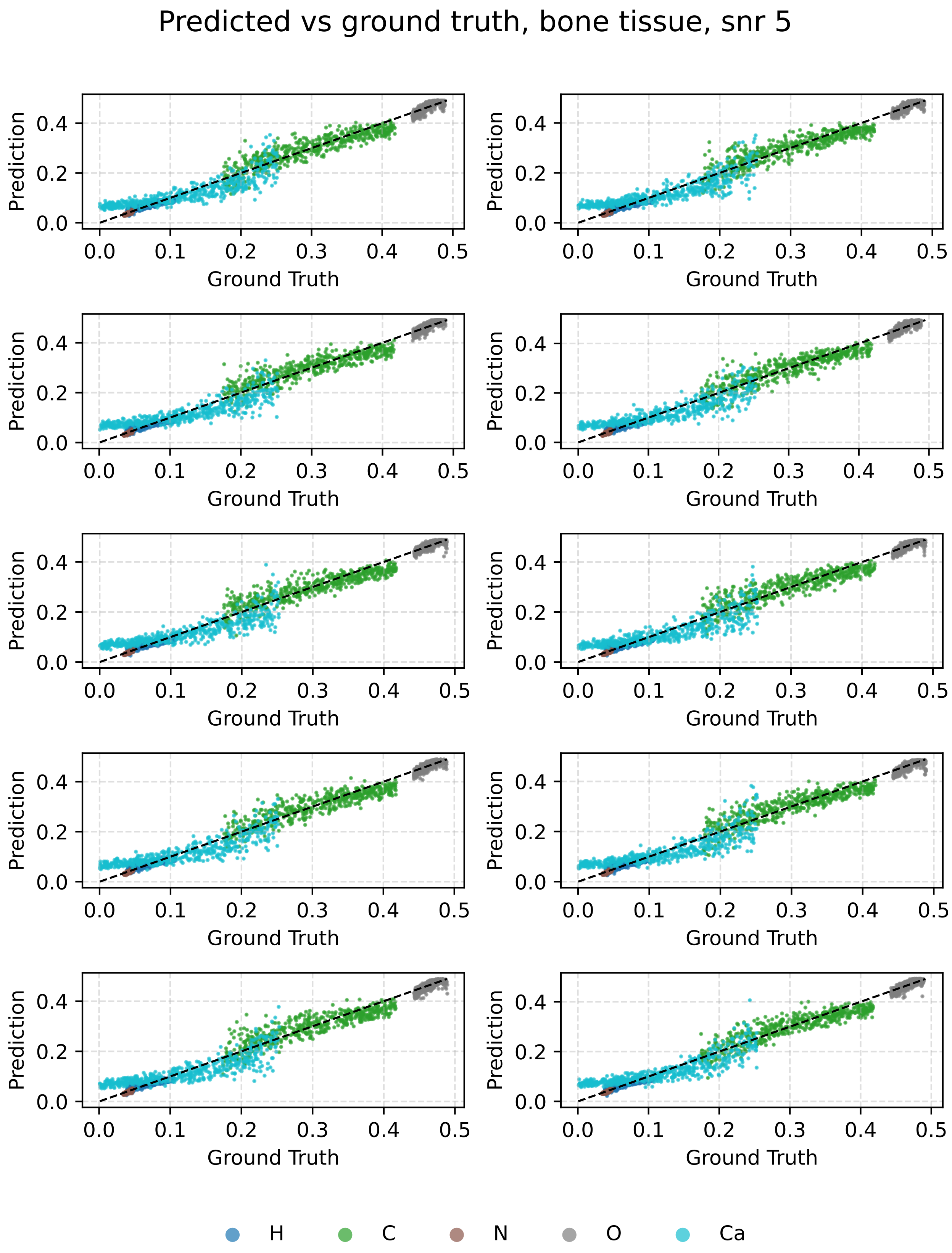

Figure 17: Same as Figure 15, but for a signal-to-noise ratio of 5.

Table 3: Weights and biases of the neural network applied to bone tissue data. The network architecture and parameter notation are illustrated in Figure 1.

		H		C		N		O		Ca		H	C	N	O	Ca
SNR	i	$w_{0,0}$	$w_{0,1}$	$w_{1,0}$	$w_{1,1}$	$w_{2,0}$	$w_{2,1}$	$w_{3,0}$	$w_{3,1}$	$w_{4,0}$	$w_{4,1}$	b_0	b_1	b_2	b_3	b_4
∞	0	8.590	-8.777	10.077	-10.237	4.827	-4.624	9.026	-8.911	-11.22	11.92	-1.311	0.110	-1.883	0.575	-0.939
∞	1	8.699	-8.837	10.194	-10.304	4.874	-4.620	9.143	-8.975	-11.11	11.86	-0.729	0.694	-1.299	1.161	-0.346
∞	2	8.941	-9.378	10.430	-10.844	5.130	-5.183	9.378	-9.517	-10.91	11.35	-0.543	0.881	-1.106	1.345	-0.159
∞	3	8.559	-9.043	10.057	-10.511	4.776	-4.869	9.001	-9.176	-11.29	11.69	-1.025	0.392	-1.595	0.858	-0.652
∞	4	8.296	-9.183	9.781	-10.636	4.525	-5.026	8.730	-9.311	-11.53	11.52	-0.362	1.057	-0.930	1.525	0.016
∞	5	8.537	-8.347	10.022	-9.813	4.776	-4.195	8.966	-8.482	-11.33	12.40	-0.816	0.606	-1.382	1.069	-0.438
∞	6	8.964	-9.029	10.467	-10.495	5.148	-4.829	9.410	-9.164	-10.83	11.65	-0.437	0.986	-1.006	1.454	-0.070
∞	7	8.513	-8.734	10.009	-10.199	4.739	-4.562	8.951	-8.867	-11.40	12.07	-0.850	0.571	-1.422	1.037	-0.469
∞	8	9.265	-9.384	10.758	-10.846	5.481	-5.202	9.700	-9.517	-10.62	11.39	-0.829	0.592	-1.400	1.057	-0.456
∞	9	8.687	-8.568	10.178	-10.034	4.876	-4.369	9.127	-8.707	-11.09	12.09	-0.785	0.639	-1.352	1.105	-0.403
10	0	-0.197	0.186	-0.211	0.191	0.088	0.295	-0.002	0.270	0.279	0.421	-1.316	0.099	-1.900	0.562	-0.868
10	1	-0.203	0.256	-0.213	0.263	0.074	0.366	-0.015	0.341	0.270	0.491	-0.725	0.692	-1.313	1.153	-0.273
10	2	0.033	-0.287	0.028	-0.283	0.304	-0.175	0.228	-0.201	0.513	-0.049	-0.545	0.870	-1.124	1.334	-0.092
10	3	-0.204	-0.085	-0.212	-0.084	0.075	0.037	-0.012	0.004	0.267	0.148	-1.034	0.376	-1.618	0.841	-0.581
10	4	-0.414	-0.241	-0.423	-0.237	-0.141	-0.124	-0.228	-0.156	0.055	0.002	-0.372	1.039	-0.958	1.503	0.077
10	5	-0.068	0.362	-0.075	0.368	0.214	0.474	0.127	0.450	0.422	0.598	-0.792	0.623	-1.377	1.090	-0.343
10	6	0.134	-0.011	0.128	-0.005	0.417	0.108	0.333	0.079	0.630	0.243	-0.455	0.961	-1.042	1.425	-0.006
10	7	-0.130	0.062	-0.142	0.066	0.151	0.176	0.063	0.148	0.352	0.299	-0.851	0.563	-1.427	1.031	-0.389
10	8	0.261	-0.138	0.254	-0.136	0.541	-0.021	0.458	-0.054	0.743	0.097	-0.839	0.580	-1.425	1.046	-0.372
10	9	-0.028	0.328	-0.037	0.332	0.246	0.442	0.165	0.416	0.446	0.557	-0.775	0.639	-1.357	1.106	-0.320
5	0	-0.144	0.204	-0.154	0.202	0.130	0.332	0.044	0.299	0.305	0.439	-1.321	0.091	-1.900	0.559	-0.852
5	1	-0.143	0.292	-0.155	0.293	0.129	0.415	0.042	0.382	0.302	0.522	-0.729	0.685	-1.318	1.150	-0.258
5	2	0.080	-0.219	0.074	-0.219	0.354	-0.096	0.273	-0.130	0.533	-0.002	-0.561	0.851	-1.147	1.318	-0.089
5	3	-0.154	-0.028	-0.165	-0.028	0.127	0.094	0.041	0.057	0.292	0.197	-1.054	0.358	-1.634	0.824	-0.579
5	4	-0.330	-0.145	-0.341	-0.145	-0.062	-0.029	-0.152	-0.067	0.115	0.070	-0.397	1.012	-0.982	1.479	0.067
5	5	-0.062	0.302	-0.067	0.302	0.210	0.425	0.127	0.388	0.399	0.531	-0.785	0.631	-1.374	1.092	-0.315
5	6	0.182	0.058	0.174	0.061	0.455	0.186	0.371	0.153	0.638	0.309	-0.481	0.933	-1.069	1.396	-0.012
5	7	-0.100	0.090	-0.110	0.086	0.170	0.227	0.085	0.184	0.351	0.336	-0.855	0.563	-1.438	1.017	-0.374
5	8	0.365	-0.043	0.354	-0.046	0.635	0.089	0.553	0.048	0.818	0.188	-0.841	0.577	-1.427	1.035	-0.358
5	9	0.014	0.358	0.009	0.356	0.275	0.482	0.200	0.449	0.446	0.587	-0.773	0.642	-1.356	1.107	-0.294

Table 4: Weights and biases of the neural network for soft tissues.

		H		C		N		O		H	C	N	O
SNR	i	$w_{0,0}$	$w_{0,1}$	$w_{1,0}$	$w_{1,1}$	$w_{2,0}$	$w_{2,1}$	$w_{3,0}$	$w_{3,1}$	b_0	b_1	b_2	b_3
∞	0	-0.771	0.836	-1.821	1.354	0.960	-0.284	1.378	-0.840	-0.480	0.578	-2.277	0.954
∞	1	-0.576	0.718	-1.647	1.241	1.140	-0.390	1.552	-0.957	-0.089	0.969	-1.884	1.338
∞	2	-0.546	0.229	-1.619	0.755	1.178	-0.893	1.589	-1.450	-0.250	0.805	-2.038	1.183
∞	3	-0.639	-0.031	-1.705	0.494	1.090	-1.156	1.505	-1.715	-0.072	0.997	-1.862	1.364
∞	4	-0.677	0.407	-1.742	0.919	1.070	-0.744	1.464	-1.280	-0.450	0.612	-2.246	0.984
∞	5	-0.259	0.796	-1.341	1.316	1.447	-0.304	1.879	-0.887	-0.238	0.828	-2.022	1.193
∞	6	-0.011	0.441	-1.094	0.979	1.703	-0.662	2.120	-1.230	-0.426	0.638	-2.216	1.010
∞	7	-0.465	0.556	-1.541	1.091	1.230	-0.528	1.667	-1.117	0.103	1.166	-1.688	1.537
∞	8	-0.013	0.089	-1.097	0.609	1.713	-1.037	2.127	-1.598	0.155	1.223	-1.635	1.591
∞	9	-0.712	1.138	-1.789	1.668	1.041	-0.005	1.428	-0.541	0.267	1.319	-1.528	1.693
10	0	-0.241	0.121	-0.491	-0.023	0.105	0.361	0.009	0.273	-0.666	0.501	-2.414	0.813
10	1	-0.214	0.175	-0.462	0.020	0.142	0.400	0.030	0.329	-0.342	0.827	-2.086	1.150
10	2	-0.013	-0.275	-0.255	-0.411	0.349	-0.060	0.251	-0.137	-0.486	0.671	-2.226	1.015
10	3	-0.241	-0.298	-0.472	-0.469	0.130	-0.067	0.010	-0.145	-0.277	0.879	-2.020	1.214
10	4	-0.068	-0.058	-0.310	-0.211	0.308	0.196	0.190	0.107	-0.738	0.422	-2.487	0.757
10	5	0.081	0.137	-0.156	-0.030	0.455	0.362	0.336	0.307	-0.330	0.849	-2.078	1.152
10	6	0.233	0.119	0.004	-0.030	0.586	0.357	0.475	0.260	-0.610	0.561	-2.357	0.879
10	7	-0.123	0.106	-0.361	-0.052	0.237	0.317	0.120	0.261	-0.154	1.020	-1.896	1.331
10	8	0.324	-0.314	0.094	-0.457	0.683	-0.098	0.562	-0.182	-0.167	1.011	-1.916	1.319
10	9	-0.011	0.354	-0.249	0.207	0.380	0.605	0.241	0.523	-0.058	1.100	-1.821	1.429
5	0	-0.219	0.080	-0.358	-0.011	-0.079	0.184	-0.083	0.198	-0.712	0.493	-2.370	0.788
5	1	-0.221	0.157	-0.343	0.059	-0.079	0.251	-0.068	0.263	-0.380	0.824	-2.044	1.127
5	2	0.047	-0.212	-0.090	-0.292	0.176	-0.114	0.187	-0.127	-0.525	0.679	-2.174	0.988
5	3	-0.199	-0.241	-0.341	-0.335	-0.058	-0.131	-0.050	-0.154	-0.321	0.877	-1.977	1.187
5	4	-0.037	-0.031	-0.174	-0.123	0.100	0.070	0.123	0.083	-0.777	0.426	-2.426	0.747
5	5	0.109	0.104	-0.030	0.006	0.250	0.196	0.240	0.196	-0.361	0.848	-2.014	1.132
5	6	0.197	0.124	0.056	0.025	0.329	0.228	0.348	0.226	-0.646	0.573	-2.311	0.850
5	7	-0.139	0.083	-0.259	-0.019	-0.014	0.182	-0.008	0.176	-0.200	1.006	-1.846	1.306
5	8	0.301	-0.275	0.171	-0.368	0.431	-0.167	0.447	-0.195	-0.215	0.995	-1.869	1.296
5	9	0.067	0.385	-0.080	0.281	0.202	0.491	0.213	0.475	-0.117	1.091	-1.777	1.392

2 Linear Model

2.1 Parameters

Parameters β_0, β_1, and β_2 of the linear model

$$\widehat{w}_i = \beta_0 + \beta_1\tilde{\mu}_1 + \beta_2\tilde{\mu}_2 + \epsilon, \tag{1}$$

where $\widehat{w}_i$ is the predicted mass fraction, $\tilde{\mu}_1$ and $\tilde{\mu}_2$ are the standardized LACs at 50 and 88 keV, respectively, and ϵ is the residual error, are listed in Tables 5 and 6.

Table 5: Parameters β_0, β_1, and β_2 of the linear model for the bone tissue.

		H			C			N			O			Ca		
SNR	i	β_0	β_1	β_2	β_0	β_1	β_2	β_0	β_1	β_2	β_0	β_1	β_2	β_0	β_1	β_2
∞	0	0.0732	0.2674	-0.28674	0.300	0.9700	-1.0395	0.0405	-0.05114	0.05481	0.465	-0.18150	0.19455	0.122	-1.0047	1.0769
∞	1	0.0732	0.2666	-0.28594	0.300	0.9671	-1.0366	0.0405	-0.05094	0.05461	0.465	-0.18102	0.19407	0.122	-1.0017	1.0738
∞	2	0.0732	0.2690	-0.28836	0.300	0.9741	-1.0436	0.0405	-0.05153	0.05520	0.465	-0.18228	0.19532	0.122	-1.0094	1.0814
∞	3	0.0732	0.2684	-0.28777	0.300	0.9719	-1.0414	0.0405	-0.05154	0.05521	0.465	-0.18147	0.19452	0.122	-1.0073	1.0794
∞	4	0.0732	0.2688	-0.28813	0.300	0.9749	-1.0444	0.0405	-0.05200	0.05568	0.465	-0.18241	0.19546	0.122	-1.0093	1.0814
∞	5	0.0732	0.2677	-0.28706	0.300	0.9683	-1.0378	0.0405	-0.05133	0.05501	0.465	-0.18134	0.19439	0.122	-1.0034	1.0755
∞	6	0.0732	0.2681	-0.28742	0.300	0.9712	-1.0407	0.0405	-0.05212	0.05579	0.465	-0.18152	0.19456	0.122	-1.0056	1.0778
∞	7	0.0732	0.2692	-0.28854	0.300	0.9762	-1.0457	0.0405	-0.05169	0.05536	0.465	-0.18271	0.19575	0.122	-1.0111	1.0832
∞	8	0.0732	0.2682	-0.28750	0.300	0.9714	-1.0409	0.0405	-0.05181	0.05548	0.465	-0.18140	0.19444	0.122	-1.0064	1.0785
∞	9	0.0732	0.2690	-0.28828	0.300	0.9723	-1.0418	0.0405	-0.05126	0.05494	0.465	-0.18250	0.19554	0.122	-1.0074	1.0796
10	0	0.0732	-0.0135	-0.00575	0.300	-0.0487	-0.0207	0.0405	0.00257	0.00109	0.465	0.00914	0.00388	0.122	0.0505	0.0215
10	1	0.0732	-0.0133	-0.00596	0.300	-0.0479	-0.0214	0.0405	0.00253	0.00113	0.465	0.00899	0.00403	0.122	0.0497	0.0222
10	2	0.0732	-0.0134	-0.00594	0.300	-0.0481	-0.0213	0.0405	0.00255	0.00112	0.465	0.00902	0.00401	0.122	0.0499	0.0221
10	3	0.0732	-0.0133	-0.00597	0.300	-0.0480	-0.0214	0.0405	0.00254	0.00113	0.465	0.00901	0.00402	0.122	0.0498	0.0222
10	4	0.0732	-0.0135	-0.00580	0.300	-0.0484	-0.0209	0.0405	0.00256	0.00110	0.465	0.00908	0.00391	0.122	0.0502	0.0217
10	5	0.0732	-0.0135	-0.00580	0.300	-0.0486	-0.0209	0.0405	0.00257	0.00110	0.465	0.00912	0.00391	0.122	0.0504	0.0216
10	6	0.0732	-0.0133	-0.00595	0.300	-0.0479	-0.0214	0.0405	0.00253	0.00113	0.465	0.00898	0.00401	0.122	0.0497	0.0222
10	7	0.0732	-0.0131	-0.00621	0.300	-0.0471	-0.0224	0.0405	0.00249	0.00118	0.465	0.00883	0.00420	0.122	0.0488	0.0232
10	8	0.0732	-0.0132	-0.00609	0.300	-0.0474	-0.0219	0.0405	0.00251	0.00115	0.465	0.00890	0.00411	0.122	0.0492	0.0227
10	9	0.0732	-0.0134	-0.00591	0.300	-0.0481	-0.0213	0.0405	0.00254	0.00113	0.465	0.00904	0.00399	0.122	0.0499	0.0221
5	0	0.0732	-0.0125	-0.00641	0.300	-0.0449	-0.0231	0.0405	0.00237	0.00122	0.465	0.00842	0.00433	0.122	0.0466	0.0239
5	1	0.0732	-0.0128	-0.00619	0.300	-0.0459	-0.0223	0.0405	0.00243	0.00118	0.465	0.00862	0.00418	0.122	0.0476	0.0231
5	2	0.0733	-0.0127	-0.00620	0.300	-0.0456	-0.0223	0.0405	0.00241	0.00118	0.465	0.00855	0.00418	0.122	0.0473	0.0231
5	3	0.0732	-0.0124	-0.00658	0.300	-0.0445	-0.0236	0.0405	0.00235	0.00125	0.465	0.00836	0.00443	0.122	0.0462	0.0245
5	4	0.0732	-0.0125	-0.00636	0.300	-0.0450	-0.0229	0.0405	0.00238	0.00121	0.465	0.00845	0.00430	0.122	0.0467	0.0238
5	5	0.0732	-0.0128	-0.00603	0.300	-0.0462	-0.0217	0.0405	0.00244	0.00115	0.465	0.00867	0.00406	0.122	0.0479	0.0225
5	6	0.0732	-0.0128	-0.00618	0.300	-0.0460	-0.0222	0.0405	0.00243	0.00118	0.465	0.00863	0.00416	0.122	0.0477	0.0230
5	7	0.0733	-0.0126	-0.00619	0.300	-0.0454	-0.0223	0.0405	0.00240	0.00117	0.465	0.00852	0.00417	0.122	0.0471	0.0231
5	8	0.0732	-0.0127	-0.00620	0.300	-0.0456	-0.0223	0.0405	0.00241	0.00117	0.465	0.00856	0.00419	0.122	0.0473	0.0231
5	9	0.0733	-0.0126	-0.00629	0.300	-0.0452	-0.0226	0.0405	0.00240	0.00119	0.465	0.00848	0.00425	0.121	0.0469	0.0235

Table 6: Parameters β_0, β_1, and β_2 of the linear model for the soft tissue.

		H			C			N			O		
SNR	i	β_0	β_1	β_2	β_0	β_1	β_2	β_0	β_1	β_2	β_0	β_1	β_2
∞	0	0.1093	-0.05882	0.054577	0.3721	-0.5819	0.3853	0.0211	0.02152	-0.01073	0.4975	0.6192	-0.4292
∞	1	0.1091	-0.05820	0.054018	0.3712	-0.5835	0.3874	0.0211	0.02118	-0.01037	0.4987	0.6205	-0.4311
∞	2	0.1093	-0.05981	0.055489	0.3717	-0.5776	0.3812	0.0211	0.02100	-0.01017	0.4978	0.6164	-0.4265
∞	3	0.1093	-0.05892	0.054583	0.3723	-0.5801	0.3833	0.0211	0.02048	-0.00959	0.4972	0.6186	-0.4283
∞	4	0.1093	-0.06027	0.056107	0.3717	-0.5831	0.3867	0.0212	0.02082	-0.01001	0.4978	0.6225	-0.4328
∞	5	0.1094	-0.05922	0.054947	0.3720	-0.5797	0.3835	0.0211	0.02108	-0.01026	0.4975	0.6179	-0.4282
∞	6	0.1093	-0.05916	0.054733	0.3722	-0.5839	0.3869	0.0211	0.02002	-0.00924	0.4974	0.6230	-0.4324
∞	7	0.1094	-0.05994	0.055823	0.3717	-0.5818	0.3846	0.0211	0.02116	-0.01029	0.4979	0.6205	-0.4301
∞	8	0.1091	-0.05770	0.053479	0.3719	-0.5868	0.3903	0.0210	0.02058	-0.00963	0.4979	0.6239	-0.4342
∞	9	0.1093	-0.06045	0.056208	0.3724	-0.5783	0.3817	0.0211	0.01966	-0.00882	0.4973	0.6191	-0.4291
10	0	0.1093	-0.00227	-0.000968	0.3721	-0.0972	-0.0582	0.0211	0.00530	0.00324	0.4975	0.0942	0.0559
10	1	0.1091	-0.00217	-0.001075	0.3712	-0.0951	-0.0574	0.0211	0.00520	0.00319	0.4987	0.0920	0.0553
10	2	0.1094	-0.00241	-0.001196	0.3721	-0.0955	-0.0611	0.0211	0.00523	0.00355	0.4975	0.0927	0.0587
10	3	0.1093	-0.00233	-0.001273	0.3721	-0.0963	-0.0617	0.0212	0.00531	0.00342	0.4974	0.0933	0.0595
10	4	0.1093	-0.00205	-0.001121	0.3717	-0.0956	-0.0589	0.0212	0.00521	0.00328	0.4979	0.0924	0.0568
10	5	0.1094	-0.00227	-0.001304	0.3723	-0.0945	-0.0636	0.0210	0.00509	0.00361	0.4973	0.0917	0.0613
10	6	0.1093	-0.00244	-0.001245	0.3727	-0.0973	-0.0625	0.0211	0.00523	0.00351	0.4969	0.0945	0.0603
10	7	0.1094	-0.00229	-0.001040	0.3719	-0.0989	-0.0592	0.0211	0.00537	0.00332	0.4976	0.0958	0.0569
10	8	0.1091	-0.00236	-0.001027	0.3721	-0.0950	-0.0605	0.0210	0.00525	0.00344	0.4977	0.0921	0.0581
10	9	0.1093	-0.00228	-0.001038	0.3715	-0.0946	-0.0591	0.0211	0.00515	0.00334	0.4981	0.0917	0.0568
5	0	0.1093	-0.00127	-0.000934	0.3720	-0.0556	-0.0441	0.0211	0.00296	0.00242	0.4976	0.0539	0.0426
5	1	0.1091	-0.00134	-0.001043	0.3712	-0.0579	-0.0463	0.0211	0.00322	0.00260	0.4986	0.0560	0.0447
5	2	0.1094	-0.00144	-0.000857	0.3717	-0.0585	-0.0423	0.0211	0.00316	0.00237	0.4978	0.0568	0.0408
5	3	0.1093	-0.00144	-0.000835	0.3724	-0.0590	-0.0398	0.0211	0.00326	0.00213	0.4971	0.0572	0.0385
5	4	0.1092	-0.00127	-0.000686	0.3708	-0.0593	-0.0405	0.0212	0.00317	0.00228	0.4987	0.0574	0.0389
5	5	0.1094	-0.00154	-0.000729	0.3724	-0.0580	-0.0409	0.0210	0.00319	0.00235	0.4972	0.0563	0.0393
5	6	0.1093	-0.00153	-0.000602	0.3723	-0.0594	-0.0397	0.0211	0.00320	0.00222	0.4972	0.0577	0.0380
5	7	0.1094	-0.00131	-0.000631	0.3725	-0.0610	-0.0395	0.0211	0.00331	0.00218	0.4970	0.0590	0.0380
5	8	0.1091	-0.00145	-0.000681	0.3721	-0.0597	-0.0380	0.0210	0.00332	0.00217	0.4978	0.0579	0.0365
5	9	0.1093	-0.00140	-0.000555	0.3712	-0.0582	-0.0377	0.0212	0.00311	0.00211	0.4984	0.0565	0.0362
